# SARS-CoV-2 variants impact RBD conformational dynamics and ACE2 accessibility

**DOI:** 10.3389/fmedt.2022.1009451

**Published:** 2022-10-05

**Authors:** Mariana Valério, Luís Borges-Araújo, Manuel N. Melo, Diana Lousa, Cláudio M. Soares

**Affiliations:** ^1^ Instituto de Tecnologia Química e Biológica António Xavier, Universidade Nova de Lisboa, Oeiras, Portugal; ^2^ Associated Laboratory LS4FUTURE, ITQB NOVA, Universidade Nova de Lisboa, Oeiras, Portugal; ^3^ iBB-Institute for Bioengineering and Biosciences, Instituto Superior Técnico, Universidade de Lisboa, Lisbon, Portugal; ^4^ Associate Laboratory i4HB—Institute for Health and Bioeconomy at Instituto Superior Técnico, Universidade de Lisboa, Lisbon, Portugal

**Keywords:** SARS-CoV-2, receptor binding domain (RBD), variants of concern (VOCs), ridge, MD simulations

## Abstract

Coronavirus disease 2019 (COVID-19), caused by the severe acute respiratory syndrome coronavirus 2 (SARS-CoV-2), has killed over 6 million people and is having a devastating social and economic impact around the world. The rise of new variants of concern (VOCs) represents a difficult challenge due to the loss of vaccine and natural immunity, as well as increased transmissibility. All VOCs contain mutations in the spike glycoprotein, which mediates fusion between the viral and host cell membranes. The spike glycoprotein binds to angiotensin-converting enzyme 2 (ACE2) *via* its receptor binding domain (RBD) initiating the infection process. Attempting to understand the effect of RBD mutations in VOCs, a lot of attention has been given to the RBD-ACE2 interaction. However, this type of analysis ignores more indirect effects, such as the conformational dynamics of the RBD itself. Observing that some mutations occur in residues that are not in direct contact with ACE2, we hypothesized that they could affect the RBD conformational dynamics. To test this, we performed long atomistic (AA) molecular dynamics (MD) simulations to investigate the structural dynamics of *wt* RBD, and that of four VOCs (Alpha, Beta, Delta, and Omicron). Our results show that the *wt* RBD presents two distinct conformations: an “open” conformation where it is free to bind ACE2; and a “closed” conformation, where the RBM ridge blocks the binding surface. The Alpha and Beta variants shift the open/closed equilibrium towards the open conformation by roughly 20%, likely increasing ACE2 binding affinity. Simulations of the Delta and Omicron variants showed extreme results, with the closed conformation being rarely observed. The Delta variant also differed substantially from the other variants, alternating between the open conformation and an alternative “reversed” one, with a significantly changed orientation of the RBM ridge. This alternate conformation could provide a fitness advantage due to increased availability for ACE2 binding, and by aiding antibody escape through epitope occlusion. These results support the hypothesis that VOCs, and particularly the Omicron and Delta variants, impact RBD conformational dynamics in a direction that promotes efficient binding to ACE2 and, in the case of Delta, may assist antibody escape.

## Introduction

Coronavirus disease 2019 (COVID-19), caused by the severe acute respiratory syndrome coronavirus 2 (SARS-CoV-2) (1–3), is a global pandemic with higher mortality than that of seasonal influenza (4). As of July 2022, over 6 million lives had been claimed by this disease (5). Infection by SARS-CoV-2 requires the fusion of viral and host cell membranes, at either the cell surface or the endosomal membrane (6). As for the severe acute respiratory syndrome coronavirus (SARS-CoV) and the Middle East respiratory syndrome-related coronavirus (MERS-CoV), the SARS-CoV-2 fusion process is mediated by the viral envelope spike (S) glycoprotein (6). Upon viral attachment or uptake, host factors trigger large-scale conformational rearrangements in the S protein, including a refolding step that leads directly to membrane fusion and viral entry (7–12).

The SARS-CoV-2 S protein is composed of a signal peptide located at the N-terminus (residues 1–13) and 2 subunits, S1 (residues 14–685) and S2 (residues 686–1,273) (13). The S1 and S2 subunits are responsible for receptor binding and membrane fusion, respectively (13). The S1 subunit consists of a N-terminal domain (residues 14–305) and a receptor binding domain, or RBD (residues 319–541). In its prefusion state, the S protein exists as a homotrimer and undergoes large conformational changes to control the exposure and accessibility of the RBD. This is done *via* an “up” and “down” mechanism, where the RBD changes from a receptor-accessible “up” conformation to a receptor-inaccessible “down” conformation (14–16).

The RBD is responsible for the interaction of SARS-CoV-2 with host cells *via* binding to the angiotensin-converting enzyme 2 (ACE2) (8, 10, 13, 17), a regulator of the renin-angiotensin system. Binding to ACE2 is one of the first steps in what is considered to be the main mode of SARS-CoV-2 viral entry, hence the importance of the RBD positional change from “down” to “up” (14, 18, 19).

**Figure 1:**
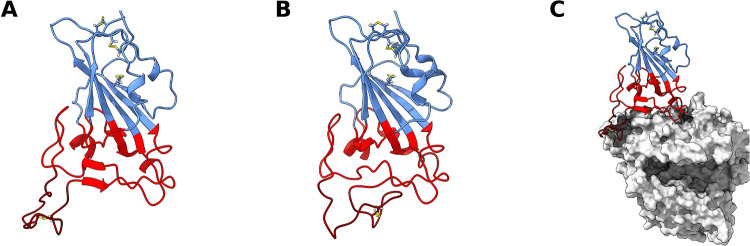
SARS-CoV-2 receptor binding domain (RBD) structure. Structure of *wt* RBD in the open (**A**) and closed (**B**) conformations. Snapshots obtained from the AA MD simulations. Disulfide bonds are represented in yellow sticks. Structure of *wt* RBD bound to ACE2 is also shown (**C**). The RBM region is colored red and the ridge in dark red, with the rest of the protein being colored in blue. ACE2 is in grey.

A lot of attention has been given to the SARS-CoV-2 RBD—ACE2 complex due to both its mechanistic implications (20–25) and pharmaceutical potential (26–32). However, not much attention has been given to the dynamics of the RBD by itself. The RBD core structure when bound to ACE2 (Figure 1A) consists of a twisted five stranded antiparallel β sheet (β1, β2, β3, β4 and β7), with short connecting helices and loops (33). While most of the S protein surface is densely glycosylated, shielding it from host defense mechanisms, the RBD itself contains only a single glycosylation site (34), N343, which is located relatively distant from the ACE2-RBD interface (Supplementary Figure S1B).

This core β sheet structure is further stabilized by 3 disulfide bonds. Between the core β4 and β7 strands (residues 438–506), there is an extended region containing 2 short β strands (β5 and β6), the alpha 4 and alpha 5 helices and loops. This region is the receptor-binding motif (RBM), which contains most of the residues that are responsible for interacting with ACE2 (14, 33). When complexed with ACE2, the RBM folds into a concave surface, that accommodates the N-terminal α-helix of ACE2, with a ridge (residues 471–491) on one side, formed by a disulfide-bridge-stabilized loop (Cys480–Cys488). It is in this surface that several RBM residues establish specific and non-specific interactions with ACE2 residues (33).

From the available experimental structural data the core β-sheet structure is very stable, but the RBM seems to be quite dynamic and not as structurally defined, unless bound to other proteins, like ACE2 (17, 33, 35–37) or antibody fragments (38–44). Molecular dynamics (MD) simulation studies have also mostly focused on RBD complexed with these proteins, and while there are MD simulation studies of the free RBD, they either focus on short simulations (45–47) or do not explore the RBM dynamics in detail (34, 45, 48–50). As such, not much is known about the conformational dynamics of this motif when unbound. This is relevant because the conformational dynamics of the SARS-CoV-2 RBD and RBM might not only play an important role in receptor recognition and binding, but also provide important information for the development of newer improved pharmaceuticals.

Recently, a significant number of naturally occurring mutations to the SARS-CoV-2 S protein have also been reported (51–54). Many of these mutations have been identified in the RBD, some of which have given rise to the dominant viral variant in certain regions due to their significant fitness advantage (51–54). Many of these RBD mutations are thought to increase fitness by increasing the binding affinity for ACE2 or by escaping neutralization by anti-SARS-CoV-2 monoclonal antibodies (55). Still, the impact of these mutations on the structural dynamics of the RBD and the RBM have not yet been investigated.

In this work, we use atomistic (AA) molecular dynamics (MD) simulation methods to investigate the structural dynamics of the SARS-CoV-2 RBD, and that of four naturally occurring variants of concern (VOCs): variant B.1.1.7, or Alpha (53) (N501Y); variant B.1.351, or Beta (51) (K417N, E484K and N501Y); variant B.1.617.2, or Delta (52) (L452R, T478K); and variant B.1.1.529, or Omicron (56, 57) (G339D, S371l, S373P, S375F, K417N, N440K, G446S, S477N, T478K, E484A, Q493R, G496S, Q498R, N501Y and Y505H). Our results show that the RBM dynamics of the *wt* RBD are such that it is not always in a conformation competent for ACE2 binding (Figure 1). Conversely, all variants, particularly Delta and Omicron, stabilize binding-competent configurations which could increase ACE2 binding efficiency. Besides impacting binding, the large conformational space visited by the variants may also hinder antibody recognition of the RBM region, thus providing a fitness advantage by facilitating antibody escape.

## Methods

### Molecular dynamics simulations

All atomistic simulations were performed with the GROMACS 2020.3 (58, 59) package and modelled using the Amber14sb forcefield (60), alongside the TIP3P water model (61). The initial *wt* RBD structure was obtained from PDB ID: 6M0J (33), which corresponds to an ACE2 bound conformation of RBD; ACE2 was excluded from this structure. The different RBD variants were generated by mutating the appropriate residues in the *wt* RBD using PyMOL (62).

It is worth noting that glycosylations were not included in our simulation systems. Despite most of the S protein surface being densely glycosylated, the RBD itself contains only a single glycosylation site far from the RBM region (34), where the dynamics reported in this work are observed. Additionally, while it has been reported that other neighboring glycosylation sites can effectively shield the RBD, this glycan shield is paired with its down-to-up conformational change in the complete S-protein: when the RBD is down the glycan shield camouflages the RBD and RBM, however, when the RBD is up it emerges from the glycan shield and presents a fully accessible RBM (34). It is in this up state, when the RBM is fully accessible to the solvent and glycans no longer play a relevant role, that the dynamics we observe may play a role in modulating binding to ACE2. Additionally, accounting for glycans would inevitably introduce degrees of freedom that would complicate sampling. Given that we do not expect glycans to play a relevant role in RBM dynamics, we opted for a reductionist approach by simulating the RBD without glycosylations.

Simulations were performed on each RBD protein structure in water. Each structure was inserted in a truncated dodecahedron box filled with water molecules (considering a minimum distance of 1.2 nm between protein and box walls). The total charge of the system was neutralized with the required number of Na^+^ ions, with additional Na^+^ and Cl^−^ ions added to the solution to reach an ionic strength of 0.1 M.

The system was energy-minimized using the steepest descent method for a maximum of 50,000 steps with position restraints on the heteroatom positions by restraining them to the crystallographic coordinates using a force constant of 1,000 kJ/mol in the X, Y and Z positions. Before performing the production runs, an initialization process was carried out in 5 stages of 100 ps each. Initially, all heavy-atoms were restrained using a force constant of 1,000 kJ/mol/nm, and at the final stage only the Cα atoms were position-restrained using the same force constant. In the first stage, the Berendsen thermostat (63) was used to initialize and maintain the simulation at 300 K, using a temperature coupling constant of 0.01 ps, without pressure control. The second stage continued to use the Berendsen thermostat but now with a coupling constant of 0.1 ps. The third stage kept the same temperature control, but introduced isotropic pressure coupling with the Berendsen barostat (63), with a coupling constant of 5.0 ps. The fourth stage changed the thermostat to V-rescale (64), with a temperature coupling constant of 0.1 ps, and the barostat to Parrinello-Rahman (65), with a pressure coupling constant of 5.0 ps. The fifth stage is equal to the fourth stage, but position restraints are only applied on Cα atoms. For production simulations, conditions were the same as for the fifth stage, but without any restraints. In all cases, 2 fs integration steps were used. Long-range electrostatic interactions were treated with the PME (66, 67) scheme, using a grid spacing of 0.12 nm, with cubic interpolation. The neighbor list was updated every twenty steps with a Verlet cutoff with a 0.8 nm radius. All bonds were constrained using the LINCS algorithm (68).

Simulations of each system were performed for at least 7 µs over 5 replicates (the *wt* was simulated for 15 µs, and the Alpha, Beta, Delta and Omicron variants for 7 µs each). The first 3 µs of simulation were considered as equilibration time and the remaining frames were used for analysis. Visualization and rendering of simulation snapshots was performed with the molecular graphics viewers VMD (69), PyMOL (62) and UCSF Chimera (70).

### Principal component analysis

PCA is a standard dimensionality reduction method that we apply here to the (3N-6)-dimensional space of possible RBD conformations (in our case, N being the number of RBD residues). PCA consists of a linear transformation that changes a set of possibly correlated dimensions into a set of linearly uncorrelated, mutually orthogonal ones, called principal components (PCs). The first PC can be defined as the direction that accounts for as much of the variance in the data as possible, with each successive PC accounting for as much of the remaining variance as possible. Reduction of data dimensionality is achieved by retaining only a few of the first PCs—which represent the strongest correlations in the data, in our case, the most important conformational motions—, thus sacrificing some information for simplicity. Discussions of the mathematical and computational backgrounds can be found elsewhere (71–74).

In this work, PCA was applied to sets of conformational coordinates obtained from MD simulations. Prior to PCA, each conformation was translationally and rotationally fitted to the RBD core Cα carbons of the *wt* crystal structure (hence the −6 in the dimensionality). PCs were determined using MDAnalysis (75), from the entire pool of simulation trajectories, considering only the coordinates of the RBD's Cα carbons. The dimensionality was reduced to the 2 most representative PCs, preserving a large part of the variance. RBD structures for each simulation frame, for each variant, could then be projected as points in this two-dimensional space, enabling a simplified visual representation of the conformation space explored by the RBD in each case.

The probability density function for each trajectory projection was estimated using a gaussian kernel estimator (73, 76) implemented in LandscapeTools’ *get_density* software as described elsewhere (73, 77). This procedure defines a probability density function *P*(*r*), with the values of *P*(*r*) being stored for the position of each data point and for the nodes of a two-dimensional uniform grid, with a mesh size of 0.5 Å. These values were used to define an energy surface, calculated as (73):

(1) $$E\left(r\right)=\;-k_{B}T\;\ln\left(\frac{P\left(r\right)}{P_{max}}\right)$$

Where *P_max_* is the maximum of the probability density function, *P*(*r*). The energy surface landscapes were analyzed by determining the energy minima and respective basins. The basins were defined as the set of all conformations whose steepest descent path along the energy surface leads to a particular minimum (73, 78, 79). Here, the steepest descent paths for each grid cell were computed, with each conformation inheriting the path of its corresponding grid cell. Landscape regions with E > 6 k_B_T were discarded, resulting in the final set of basins for each data set.

### Residue interaction network analysis

Residue interaction networks (RINs) are graph representations of protein structures, where the nodes represent amino acid residues, and the edges represent interactions between residues. Pairwise residue interactions were analyzed for the 5,000 lowest energy conformations obtained for the most populated open, closed and reversed conformation basins of the energy surface landscapes of each RBD variant, using RIP-MD (80). Several types of interactions between AAs were probed: Cα contacts, hydrogen bonds, salt bridges, disulfide bonds, cation-π, π-π, Arg-Arg, Coulomb and van der Waals. The parameters defining each interaction, as well as their mathematical formulation can be found elsewhere (80). Once the interactions were determined, the interaction networks were visualized using Cytoscape (81).

## Results and discussion

Our aim was to study the conformational dynamics of the SARS-CoV-2 RBD, as well as that of several other SARS-CoV-2 VOCs in solution. To this effect, we simulated the *wt*, Alpha, Beta, Delta and Omicron variants of the SARS-CoV-2 RBD. The Gamma variant was not studied due to its similarity to the Beta variant: in the RBD both variants share the E484K and N501Y mutations; the single difference is the K417N mutation in the Beta variant vs. K417T in Gamma (82, 83). In either case, K417 is mutated to a residue with a polar uncharged side chain, which should impact the interaction network similarly. As such, the conformational dynamics specific to the RBD and RBM are expected to be similar.

### *Wt* RBD presents two distinct RBM conformations in aqueous solution

**Figure 2:**
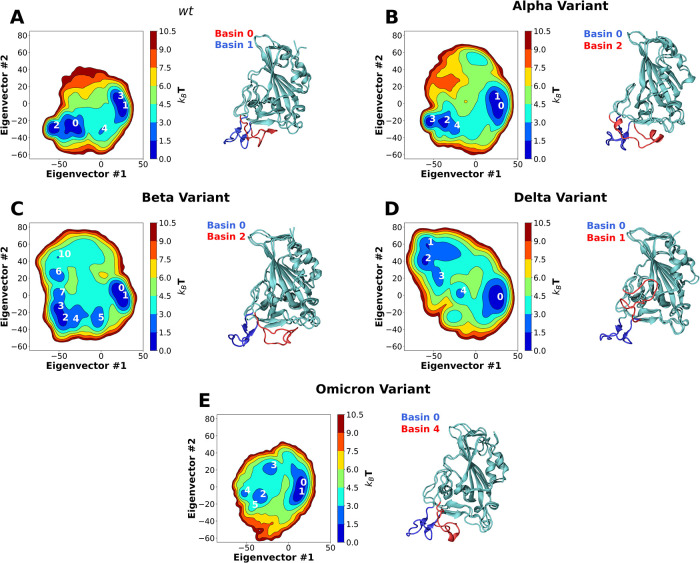
Two-dimension principal component analysis (PCA) of SARS-CoV-2 RBD conformational dynamics in water. Plots of the first two principal components determined from the Cα backbone of the *wt* RBD (**A**) as well as the Alpha (**B**), Beta (**C**), Delta (**D**) and Omicron (**E**) variants. Basins with k_B_T < 3 are numbered in each figure. Snapshots of the lowest energy structures for selected open and closed basins are also shown. The ridge regions of the open and closed snapshots are colored in blue and red, respectively.

Visual inspection of the trajectories obtained in the simulation of *wt* RBD in water revealed that large dynamic conformational changes occur in the RBM region (Figure 2A; Supplementary Video S1). The dynamics observed appear to show an opening and closing of the ACE2 binding surface of the RBM. To better characterize these conformational dynamics, we performed principal component analysis (PCA) on the coordinates recovered from these simulations, reducing them to 2 principal components; this 2D configuration space sampling was expressed as free energy landscapes (Figure 2).

For *wt* RBD, we observe two deep basin clusters (Figure 2A), as well as several other lesser populated basins. Closer analysis of the RBD conformations that make up each basin shows that *wt* basins 1 and 3 correspond to conformations close to the ACE2-bound one determined by x-ray crystallography (33) (Figures 1A, 2A). We named these “open” configurations. In contrast, the second basin cluster (basins 0 and 2) was made up by conformations quite distinct from the open ones. In these basins, the loop that makes up the RBM is twisted and collapsed over the region that binds ACE2, effectively hiding it from the solvent (Figures 1B, 2A). We named these conformations “closed”. Further analysis of the PCA results reveals that the *wt* RBD is in a closed state for more than half of the simulation time (∼55.5%, Supplementary Table S1). Given that in these conformations the RBM closes on itself, hiding the ACE2 binding surface, we can speculate that the RBD would be unable to effectively bind to ACE2 and initiate an ACE2-dependent infection process. Moreover, the open and closed states were visited reversibly (Supplementary Figure S2), indicating that our simulations were not kinetically trapped in either basin. The open and closed RBD conformations reported here should not be confused with the “up” and “down” S protein conformational states which control the exposure of the RBD in the context of the S protein homotrimer. The open/closed dynamics likely act as an additional RBM exposure control, which would be particularly important for RBDs in the “up” S protein conformational state where they are fully exposed to the solvent.

Residue interaction network (RIN) analysis was performed for the 5,000 lowest energy structures of basins 1 (open) and 0 (closed). From the identified interactions, we selected those that were present in over 50% of the simulation frames (Supplementary Figure S4). We also only considered interactions that are established by RBM residues, or those in their immediate vicinity. These RINs were then used to probe the different intramolecular interactions established in each of the conformations.

**Figure 3:**
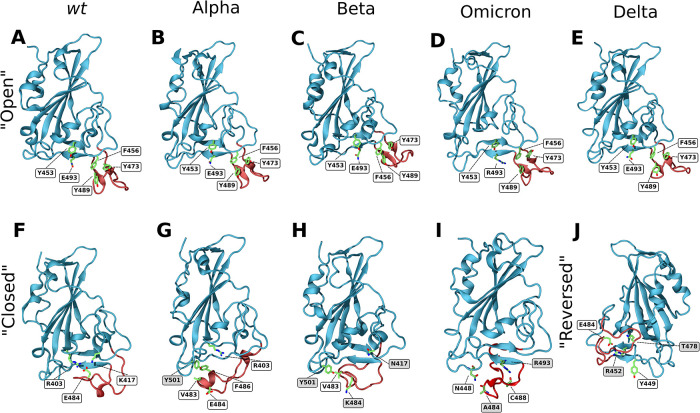
Closeup snapshots of SARS-CoV-2 RBD intramolecular interactions that stabilize the various conformations. Snapshots from AA MD simulations showcasing crucial intramolecular interactions responsible for stabilizing the open, closed, and reversed conformations for the *wt* (**A,F**), Alpha (**B,G**), Beta (**C,H**), Omicron (**D,I**) and Delta (**E,J**) RBD variants. The ridge region of the RBD is colored in red and residues of interest in green. Text labels indicate relevant residues, with shaded labels indicating mutations relative to *wt*. All figures are rotated 180° relative to Figures 1, 2, apart from snapshot J.

In the open conformation, the RBD ridge is stabilized by a triple π-stacking interaction between residues Y489–F456–Y473 and a hydrogen bond between Y489–Y473. Additionally, two hydrogen bonds are established between residues Y453 and E493, which help stabilize the formation of a small β-sheet (Figure 3A).

In the closed conformation, however, the π-stacking interactions are broken, and new interactions with RBD core residues are formed in their place. F456 forms a stable π-stacking with Y421, Y489 forms a transient π-stacking interaction with F486 and Y473 forms a hydrogen bond with the backbone of Y451. Moreover, E484 forms a salt bridge with R403, that is found in the RBD core, and a hydrogen bond with K417 (Figure 3F). This hydrogen bond does not show up in the RIN, as K417 can establish a bond with each of the two glutamate oxygens, each with ∼40% prevalence (each thus below our 50% selection cutoff). These two interactions, together with the formation of three hydrogen bonds (C480–S494–G482–Q493) are responsible for the closing of the ridge and consequent shielding of the ACE2 binding surface. The importance of the E484–R403 and E484–K417 interactions for the closing of the loop was confirmed by simulating the E484K and K417N mutants. Either of these single mutations were enough to completely deplete the closed conformation (Supplementary Figures S3A,B for E484K and K417N, respectively). This shows that both these interactions are crucial for the stabilization of the *wt* closed state. Still, several other transient hydrogen bonds, formed between residues L492, G493 and S494 of strand β6, and T478, C480, N481, G482 and E484 of the RBM ridge, assist in stabilizing the structure.

The closed conformation does not seem to substantially impact the RBD's secondary structure (Supplementary Figure S9). The largest impact appears to be limited to residues 473–474 and 488–489, that in the open state display a slight β-sheet character. However, upon closing, this β-sheet character disappears. This effect comes from residues 473 and 489 no longer participating in the triple π-stacking that was likely stabilizing this region.

Apart from impacting ACE2 accessibility, the closing of the RBM ridge also decreases the solvent accessible surface area (SASA) of the RBD by slightly over 3% (Supplementary Table S2).

Although other studies have noted the high flexibility in the RBM region of the RBD (45–47, 84), this is, as far as we know, the first report of this hinge mechanism that can effectively hide the ACE2 binding surface of the RBD from binding partners, which could only be observed through the analysis of µs-long MD simulations. While it is likely that induced fit interactions might assist in opening a closed conformation for binding to ACE2, it is safe to assume that the closed conformation will have its binding to ACE2 substantially hindered when compared to an open conformation. Other studies have also observed RBM concealment mechanisms in the context of the spike protein (84). It has been shown that “down” state RBDs can conceal their RBM by interacting with the neighboring RBDs, in a temperature dependent manner. This further showcases the tendency of the RBM to conceal its hydrophobic surface, either by closing in on itself (as observed in the present study) or by interacting with neighbouring RBDs [as observed by Rath et al. (84)].

### SARS-CoV-2 alpha and beta variants impact RBM conformational dynamics and exposure

The first SARS-CoV-2 variant of concern to be identified was first detected in the UK. It is often referred to as B.1.1.7 or Alpha variant and has only one mutation in the RBD region—N501Y. A second variant emerged soon after in South Africa, independently of B.1.1.7, referred to as B.1.351 or Beta variant. In the RBD region, this variant shares the N501Y mutation with the Alpha variant and includes two others: K417N and E484K (53).

In line with what was observed for the *wt* RBD, MD simulations of the RBDs from the Alpha and Beta variants also showed the prevalence of two sets of RBM conformations, corresponding to open and closed conformations (Supplementary Videos S2, S3). PCA analysis of the Alpha variant trajectory shows two deep basin clusters (Figure 2B), basins 0 and 1, and basins 2 and 3, which correspond to open and closed conformations respectively. However, unlike the *wt* variant, the Alpha variant remains most of the simulation time in an open conformation (∼72.64%, Supplementary Table S1). The Beta variant (Figure 2C) also has two deep basin clusters (basins 0 and 1, and basins 2 and 3), corresponding to open and closed conformations, respectively. Like the Alpha variant, Beta remains in an open conformation for substantially longer time than the *wt* (∼69%, Supplementary Table S1). In both cases, and as for *wt*, our simulations were able to reversibly visit both states (Supplementary Figure S2).

Both Alpha and Beta variants shift the open/closed equilibrium towards more open conformations by roughly 20%. An opening ΔΔG was calculated from the ratio between the time spent in the open and closed states, where the time spent in each individual open and closed basin was added together (Supplementary Table S1). The equilibrium shift led to a decrease in the opening ΔΔG from 0.55 ± 0.17 kJ/mol, in the case of *wt* RBD, to −2.44 ± 0.22 and −2.09 ± 0.14 kJ/mol, for the Alpha and Beta variants, respectively. As mentioned previously, it is likely that only the open conformations are fully available to bind to ACE2, meaning that these mutations substantially increase the accessibility of RBD to ACE2, and probably impact ACE2-RBD binding.

By analyzing the intramolecular residue interactions for both variants, we observe that the interactions which stabilize the open conformation in the *wt* RBD are conserved in both Alpha and Beta variants, namely the triple π-stacking between residues Y489–F456–Y473, as well as the hydrogen bond between Y489 and Y473. An additional hydrogen bond between Q493 and Y453 assists in stabilizing the β6 strand (Figures 3B,C).

Interestingly, in both the open and closed conformations of the Alpha variant, the interactions established by residue Y501 (Alpha's only mutation in the RBD) that were previously present in the *wt* variant are maintained in the Alpha variant (two hydrogen bonds established through the residue backbones: Q458–Y501 and Y501–Q506). However, the main interactions that stabilize the closed conformations differ between the Alpha variant and *wt* (although some transient hydrogen bonds between strand β6 and the RBM ridge do remain). Instead of the E484–R403 salt bridge seen for *wt*, in the Alpha variant the closed conformation is promoted by the formation of hydrophobic interactions between the mutated Y501, V483 and F486 (Figure 3G). This arrangement hinders the establishment of the E484–R403 salt-bridge (as can be seen in Supplementary Video S2) while being itself less stable than the open conformations. This is the likely cause for the decrease in percentage of closed state observed for Alpha. Progression to the E484–R403 salt-bridge may also be prevented in part by the establishment of a short α-helix, discussed ahead.

In the Beta variant, the closed conformation is notably impacted by both the E484K and the N501Y mutations. The E484K mutation prevents the formation of the E484–R403 salt bridge that was crucial for the stability of the closed conformation in the *wt* protein. However, unlike the single E484K mutant (Supplementary Figure S3), the Beta variant can still reach a closed conformation. This is because it can establish the same hydrophobic interaction between Y501 and V483 as the Alpha variant (Figure 3H). This closed state is also stabilized by the same transient hydrogen bonds between strand β6 and the RBM ridge seen in the *wt* and Alpha variants.

Concerning the secondary structure, there are no substantial differences between the Alpha or Beta open states and the *wt* open state (Supplementary Figure S9). However, upon closing, both Alpha and Delta form a small α-helix between residues 475 and 490, for roughly 30% of the simulation time. This helical character might be relevant for the Alpha variant, as it assists in facing the E484 sidechain away from R403 (Figures 3G,H), hindering the formation of the salt-bridge. Additionally, the Alpha variant also shows some helicity in residues 482–489, which likely arises from contacts between residues in this helix and the mutated N501Y.

Curiously, while the Alpha variant also shows a considerable decrease in SASA upon closing (∼5%), the Beta variant shows no substantial change.

Overall, these results showcase a possible alternative mechanism for how the Alpha and Beta variants might facilitate viral entry into the host cells. By shifting the open/closed equilibrium towards the ACE2-accessible open conformation, both variants are facilitating ACE2–RBD binding, which will inevitably lead to an increase in binding affinity and enhanced receptor-dependent infection.

### SARS-CoV-2 delta variant shows conformational dynamics distinct from the other variants

During the second half of 2021 the global dominant SARS-CoV-2 variant was B.1.617.2 (or Delta) (52). It contains two mutations in the RBD region: L452R and T478K. Like the *wt*, Alpha and Beta variants, MD simulations of the Delta RBD show the prevalence of two sets of RBM conformations, one of which corresponds to the *wt* open conformation (Supplementary Video S4) and is stabilized by the same interactions observed for the three other variants (Figure 3E). However, unlike those variants, MD simulations of the Delta RBD do not show the occurrence of a closed conformation at all. Instead, an alternative open conformation is present, which we refer to as “reversed”. PCA analysis of the Delta variant trajectory, shows two deep basins, 0 and 2 in Figure 2D, which correspond to the open and reversed conformations, respectively. Similarly to the other variants, simulations were able to reversibly visit the two states (Supplementary Figure S2).

The reversed conformation showcases the incredible flexibility of the RBM region, which not only opens and closes over the ACE2 binding surface of the RBD but acts as a two-way hinge that leans to the side of the RBD. This alternative conformation might also prove significant advantages over the *wt* open state: RBD-targeting antibodies are known to bind *via* recognition of the RBM ridge region (22, 85); the reversed state putatively hides this region from antibody recognition, while still providing an open ACE2 binding surface for infection.

A hydrogen bond between the mutated R452 on strand β5 and Y449 appears to be one of the main driving forces folding the Delta variant's ridge region backwards. This interaction destabilizes the β5 strand and enables the ridge to move up and interact with the core. Transient interactions between ridge residues G476, S477 as well as the mutated K478 with residues R346, F347 and N354 of strand β1 stabilize the contact between the ridge loop and the RBD core, keeping it locked in place (Figure 3J).

Regarding the secondary structure, much like the other variants, the Delta open conformation is very similar to that of the *wt* (Supplementary Table S3). However, as expected, the reversed conformation shows substantial differences. In this state, the two small beta strands formed by residues 473–474 and 488–489, present in the open conformation, are completely lost. Additionally, the beta-sheet formed by strands β5 and β6 becomes less prevalent, likely due to the L452R mutation (one of the β5 strand residues that destabilizes the β-sheet by establishing a new interaction with Y449). Curiously, like in the Alpha and Beta variants, there is also a significant alpha helical character between residues 490 and 475.

As for the closed conformations of the *wt*, Alpha and Beta variants, the Delta reversed conformation also leads to a decrease in SASA (∼3%). Unlike the closed conformations, however, this alternative open conformation still presents a fully accessible ACE2 binding surface.

### SARS-CoV-2 omicron variant further improves RBM accessibility compared to alpha and beta

Towards the end of 2021 a new VOC—B.1.1.529 or Omicron—overtook Delta as the dominant variant in most world regions (86). The Omicron variant is highly distinct from other VOCs (87), containing 15 mutations in the RBD region (G339D, S371l, S373P, S375F, K417N, N440K, G446S, S477N, T478K, E484A, Q493R, G496S, Q498R, N501Y and Y505H), 10 of which are concentrated in the RBM (N440K, G446S, S477N, T478K, E484A, Q493R, G496S, Q498R, N501Y and Y505H). Some of these mutations are also observed, or are similar to those, in the Alpha, Beta and Delta variants: K417N, T478K, E484A and N501Y.

Unlike the other variants, MD simulations of the Omicron RBD do not show a clear prevalence of two distinct sets of RBM conformations (Figure 2E; Supplementary Video S5). While PCA analysis shows a deep basin cluster corresponding to the *wt* open conformation, there are only shallow basins corresponding to the closed conformation. No alternative reversed conformation is observed. The open conformation accounted for almost the entire set of configurations sampled during the simulations of the in Omicron variant (∼95%, Supplementary Table S1), substantially larger than that of the *wt*, Alpha or Beta variants. The simulations were able to reversibly visit either state (Supplementary Figure S2). When compared to the *wt* variant, Omicron resulted in a 50% shift in the open/closed equilibrium towards more open conformations, with an opening ΔΔG of −8.38 ± 0.5 kJ/mol.

The triple π-stacking interactions and hydrogen bonds that stabilize the open conformation in the Omicron variant are common to those present in the *wt*, Alpha, Beta and Delta variants (Figure 3D). However, the closed conformation is quite distinct from the one seen with the other variants (Figure 3I). Only two transient hydrogen bonds—one between the sidechain amide of N448 and the backbone of A484, and the other between the sidechain of R493 and the backbone of C488—are in place to stabilize the closed conformation. This contrasts with the stronger interactions present in the *wt*, Alpha and Beta closed conformations. The *wt* closed conformation was stabilized by the formation of two salt bridges between K417, E484 and R403. In Omicron, E484 is mutated to an alanine preventing these two interactions. In the Alpha and Beta variants, instead of these salt bridges, several hydrophobic interactions between Y501, V483 and F486 promoted the closing of the loop. The Omicron Y501N mutation disrupts this hydrophobic core. It is clear that several mutations to the Omicron RBM actively hinder the closing of the loop while promoting the open conformation, which ultimately facilitates ACE2-RBD binding.

No substantial differences in secondary structure between the Omicron and *wt* variants in either the open or closed states were observed (Supplementary Figure S9).

It is worth noting that the Omicron variant has recently been classified into five different lineages based on their mutations, BA.1, BA.2, BA.3 (56, 88, 89), BA.4 and BA.5 (90). The BA.1 lineage was used in this work. Regarding the RBD region, all five lineages have 11 mutations in common (G339D, S373P, S375F, K417N, N440K, S477N, T478K, E484A, Q498R, N501Y and Y505H). BA.1 has three specific mutations (S371l, G446S and G496S), BA.2 has four (S371F, T376A, D405N and R408S), BA.3 has a combination of BA.1 and BA.2 mutations (G446S, S371F and D405N) and BA.4 and BA.5 have several mutations in common with BA.2 (S371F, T376A, D405N and R408S), plus two others (L452R and F486V).

According to our simulations, the mutations most associated with the open/closing dynamics of the RBD ridge are, for the most part, common to the BA.1, BA.2, BA.3, BA.4 and BA.5 lineages (E484A, Q493R, N501Y, and K417N). The only exception to this is the Q493R mutation in the BA.4 and BA.5 lineages, which does not occur and instead the *wt* Q493 residue is maintained. Additionally, the mutations specific to the BA.2 and BA.3 lineages are relatively far from the RBM loop region and as such are unlikely to play a role in this mechanism (Supplementary Figures S16B,C). Two of the mutations specific to BA.1 are present in this region (G446S and G496S, Supplementary Figure S16A). However, these mutations did not play an obvious role in the RBD ridge dynamics, and we speculate that reverting these residues back to glycines would not impact these dynamics significantly. Taking all of this into consideration, we expect that the RBD dynamics of the other Omicron lineages to be fairly similar to that of BA.1.

### Impact of SARS-CoV-2 variants on ACE2 binding affinity

To find experimental basis for our results, we compiled ACE2-RBD binding kinetics data from recent studies (91–102) (Supplementary Table S3). These results were obtained by surface plasmon resonance (SPR) and biolayer interferometry (BLI) and encompass data regarding both the *wt* and studied variants. Additionally, we compiled results obtained for just the RBD as well as for the entire S protein. While the binding kinetics values recovered from these studies are not fully consistent with each other, likely due to differences in particular experimental setups, they are mostly in the same range, and appear to follow similar trends. Regarding the equilibrium dissociation constant (*K_d_*), all variants have an increased binding affinity when compared to the *wt*. However, with the currently available data, it is hard to distinguish between the efficiency of the several variants, with the Alpha and Beta variants showing a slightly better affinity than Delta.

To get more information, we analyzed both the association (*k_on_*) and dissociation rate constants (*k_off_*). *k_off_* reflects the lifetime of the protein-protein complex and as such, the strength of the interaction. We observe a consistent decrease in *k_off_* for the variants in comparison to the *wt*. The Alpha and Beta variants stand out from Delta and Omicron in this regard, with substantially lower *k_off_* values. These results hint at the VOCs interacting more strongly with ACE2 than the *wt*, with the Alpha and Beta complexes being substantially more stable than those of Delta and Omicron. Several other MD studies have studied the impact of these mutations on ACE2-RBD contacts, binding affinity and binding modes, showcasing how the substantially altered ACE2-RBD interaction of the Alpha and Beta variants might be outperforming that of the *wt* variant (103–108). The Delta variant does not contain mutations to the RBD ACE2 binding surface and, as such, the interactions established are not substantially different from those of *wt*. This is reflected in a *k_off_* that is closer to, if still lower than, that of the *wt*.

The variants also substantially impact *k_on_*. This rate constant reflects the efficiency with which protein–protein collisions lead to a bound state. While a couple of studies show no significant impact (91, 95), most show that the variants lead to a substantial increase in *k_on_*, reflecting an increase in RBD accessibility to ACE2 (92–94, 96, 101). We propose that this can be explained by the significant changes in RBM conformational dynamics that we have here described, where mutations lead to a decrease in prevalence of the closed state, favoring binding. As such, our results point to an alternative mechanism for enhancing RBD-ACE2 binding, not by directly strengthening ACE2-RBD interactions, but rather by boosting, *via* modulation of ridge dynamics, the ACE2 binding competence.

## Conclusion

In this work we performed AA MD simulations of the SARS-CoV-2 RBD, as well as that of the Alpha, Beta, Delta and Omicron VOCs, to characterize the impact of the mutations on RBD conformational dynamics in solution. Our results show that the *wt* RBD adopts two distinct conformations in equilibrium: an open conformation where the RBD is free to bind ACE2; and a closed conformation, where the RBM ridge blocks the ACE2 binding surface and likely hinders binding to ACE2. We characterized the two states and showed that they originate from specific intramolecular interactions between residues of the RBM ridge and those of the surface that binds ACE2. As far as we know, this is the first report of this “hinge-like” mechanism, which can effectively shield the surface of RBD binding ACE2 from the solvent and binding partners. This mechanism is yet to be seen in experimentally solved RBD structures, which have thus far struggled to fully resolve the unbound RBM region (14, 25, 109). The RBM is found unresolved in most structures due to the large flexibility of the region, and those that are fully resolved are often structures of RBD complexed with either ACE2 (17, 33, 35–37), antibodies (38–44) or itself by dimerizing *via* the surface binding ACE2 (110, 111).

The four variants tested in this work, significantly impacted the open/closed equilibrium we observed for *wt* RBD. Both Alpha and Beta variants shifted the equilibrium towards more open conformations by roughly 20%. In Omicron the open conformation accounted for 96% of simulation time while the Delta variant did not show the presence of a closed conformation at all. This shift towards more open conformations likely enhances ACE2 binding affinity by increasing accessibility to the RBM and facilitating binding. Several experimental binding studies have shown that these variants lead to a substantial increase in ACE2-RBD binding association rate constant, reflecting an increased ACE2 accessibility, in agreement with our findings.

Additionally, the Delta variant showed an alternative open conformation, distinct from that of the other variants. This alternative conformation keeps the ACE2 binding surface open and accessible for binding, but significantly alters the conformation of the RBM ridge. This state presents a substantially altered ridge region, which bends backwards towards the RBD core, shielding some of it from exposure. We hypothesize that this may provide a fitness advantage by aiding in antibody escape, since many RBD-targeting antibodies bind to the RBM ridge region (39, 85, 112, 113). These RBD-targeting antibodies are also more sensitive to viral evolution than antibodies that bind other regions of the RBD (114). In the alternative open conformation, the ridge may be not as easily recognized, while the ACE2 binding surface remains unobstructed for infection. The substantially different conformational dynamics of the RBM region between the variants, correlates well with the hypothesis proposed by Quaglia et al. that mutations are enriched at intrinsically disordered regions of the SARS-CoV-2 proteome and that they may contribute towards immune evasion (115).

These results show that the mutations found in the four VOCs studied impact RBD conformational dynamics in a direction that promotes efficient binding to ACE2 and (in the case of the Delta variant) antibody escape, an effect which has thus far been disregarded. In this context, our findings can also help explain some of the antibody-evading characteristics of the emergent Omicron variant.

## Data Availability

The raw data supporting the conclusions of this article will be made available by the authors, without undue reservation.
