# Supplementary material for: SARS-CoV-2 variants impact RBD conformational dynamics and ACE2 accessibility
Source: Front Med Technol. 2022 Oct 5;4:1009451. doi: 10.3389/fmedt.2022.1009451 (PMC9581196; doi:10.3389/fmedt.2022.1009451)
Supplement: Supplementary file 6 [file DataSheet1.pdf]

Supplementary Material

Videos S1 to S5. RBD and RBM conformation dynamics for the *wt*, alpha, beta, delta and omicron variants. Trajectory samples recovered from the AA MD simulations of the *wt*, alpha, beta and delta RBD in water. The ridge region of the RBD is colored in red. Residues of interest are labelled at the start of the video. Renderization done with VMD, with the positions averaged over 5 consecutive frames.

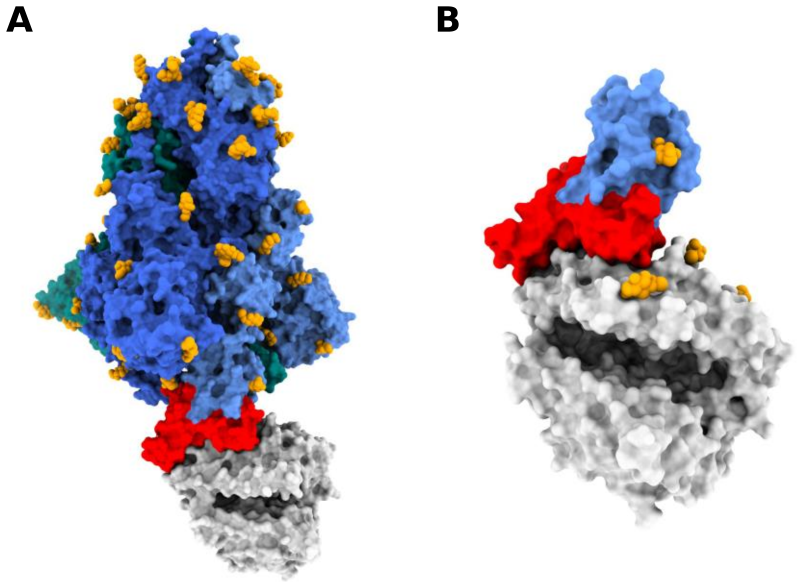

Figure S1. ACE2 bound to glycosylated SARS-CoV-2 S protein. (A) ACE2 (White) bound to SARS-CoV-2 S glycoprotein (blue). (B) Closeup of ACE2 bound RBD. RBM is rendered in red. Glycosylations are rendered in orange.

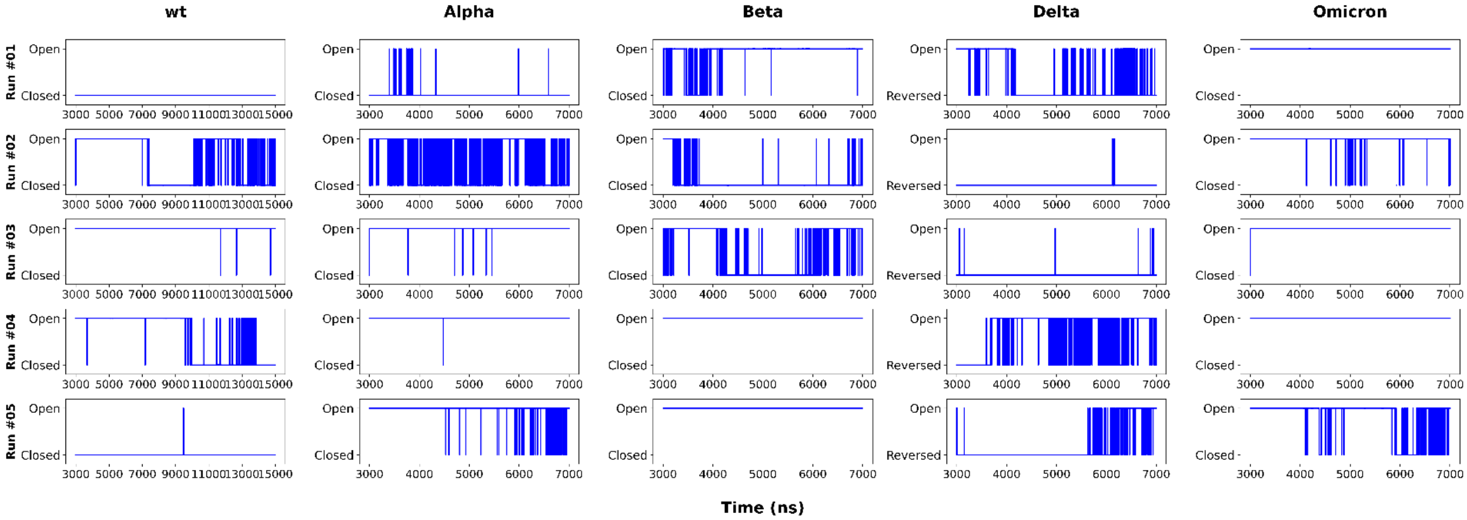

Figure S2. RBD open – closed dynamics over simulation time. RBD open – closed dynamics as determined by analysis of the conformational basins (Supplementary Table S1) recovered by PCA. Data shown for the five replicas for each variant tested. The first 3 µs of simulation were used for equilibration.

Table S1. Energy surface landscape analysis from 2D PCA of SARS-CoV-2 RBD conformational dynamics in water. Energy surface landscape analysis and defined basins for each of the tested RBD variants. Energy minima, frame percentage and loop conformation for each of the basins is also given. Overall analysis of "open" vs. "closed" conformation is also shown. 95 % Confidence intervals (CI) were calculated with bootstrap resampling from the frame percentages recovered from the individual simulation replicas. Representative structures can be seen in figures S8, S9, S10 and S11.

	Basin	Free Energy	$\langle E \rangle / k_BT$	S/R	E_{min}/k_BT	Frame percentage (%)	Loop Conformation	Time in closed state (%) ± CI (95%)	Time in open state (%) ± CI (95%)	opening ΔΔG (kJ/mol)
	0	-5.16	1.36	6.52	0.44	37.69	Closed			
	1	-4.67	0.99	5.67	0.26	23.01	Open			
	2	-4.41	0.57	4.98	0.00	17.66	Closed			
	3	-4.20	1.04	5.24	0.44	14.42	Open			
	4	-3.28	3.62	6.90	2.86	5.77	Open			
WT	5	-1.57	4.07	5.64	3.61	1.04	Open	$55.50^{+2.12}_{-2.93}$	$44.49^{+0.93}_{-1.12}$	$0.5^{+0.21}_{-0.13}$
	6	0.79	5.30	4.51	5.02	0.13	Open			
	7	0.83	5.42	4.60	5.21	0.14	Closed			
	8	0.91	5.39	4.48	5.01	0.12	Open			
	9	2.70	5.06	2.37	5.01	0.01	Closed			
					**Total**	99.99				
	0	-5.20	1.02	6.22	0.00	39.18	Open			
	1	-4.64	1.47	6.11	0.35	22.41	Open			
	2	-4.37	1.84	6.21	0.83	17.23	Closed			
	3	-3.82	0.96	4.78	0.35	9.85	Closed			
	4	-3.10	2.77	5.86	1.83	4.82	Open			
Alpha	5	-2.71	3.88	6.58	3.01	3.28	Open	$27.31^{+1.79}_{-2.04}$	$72.64^{+1.16}_{-1.43}$	$-2.44^{+0.21}_{-0.23}$
	6	-2.34	4.10	6.44	3.11	2.41	Open			
	7	-0.28	5.21	5.49	4.93	0.29	Open			
	8	-0.11	4.43	4.54	4.05	0.25	Open			
	9	0.11	5.46	5.35	5.10	0.23	Closed			
					**Total**	99.95				
	0	-4.95	0.58	5.53	0.00	23.73	Open			
	1	-4.70	0.46	5.16	0.09	18.41	Open			
	2	-4.38	1.31	5.69	0.90	13.43	Closed			
	3	-3.98	1.32	5.30	0.90	9.03	Closed			
	4	-3.83	2.70	6.53	1.93	7.72	Closed			
	5	-3.78	2.86	6.64	2.02	7.36	Open			
	6	-3.36	2.93	6.30	2.30	4.86	Open			
Beta	7	-3.20	2.20	5.40	1.68	4.14	Open	$30.18^{+1.24}_{-1.74}$	$69.81^{+0.66}_{-0.73}$	$-2.09^{+0.13}_{-0.15}$
	8	-2.93	4.08	7.02	3.32	3.21	Open			
	9	-2.71	3.66	6.37	3.11	2.54	Open			
	10	-2.58	3.53	6.12	2.98	2.23	Open			
	11	-2.14	3.70	5.85	3.28	1.44	Open			
	12	-1.74	3.78	5.52	3.48	0.95	Open			
	13	-1.72	3.99	5.71	3.70	0.94	Open			
					**Total**	99.99				
	0	-5.74	1.03	6.77	0.00	53.51	Open			
	1	-4.97	2.32	7.29	1.46	24.76	Reverse Open			
	2	-4.13	1.70	5.84	1.20	10.75	Reverse Open			
Delta	3	-3.46	3.17	6.63	2.62	5.50	Open	N/A	100.00	N/A
	4	-2.93	3.34	6.27	2.63	3.22	Open			
	5	-1.88	4.15	6.03	3.77	1.12	Open			
	6	-1.85	4.12	5.97	3.60	1.13	Open			
					**Total**	99.99				
	0	-4.77	1,33	6,11	0,17	37,27	Open			
	1	-4.71	0,88	5,59	0,00	35,10	Open			
	2	-3.78	3,06	6,83	1,84	13,87	Open			
Omicron	3	-3.49	3,12	6,61	2,11	10,35	Open	$3.35^{+0.33}_{-0.55}$	$96.59^{+2.01}_{-2.22}$	$-8.38^{+0.50}_{-0.29}$
	4	-2.19	3,12	5,31	2,27	2,83	Closed			
	5	-0.46	4,37	4,83	3,89	0,52	Closed			
					**Total**	99,94				

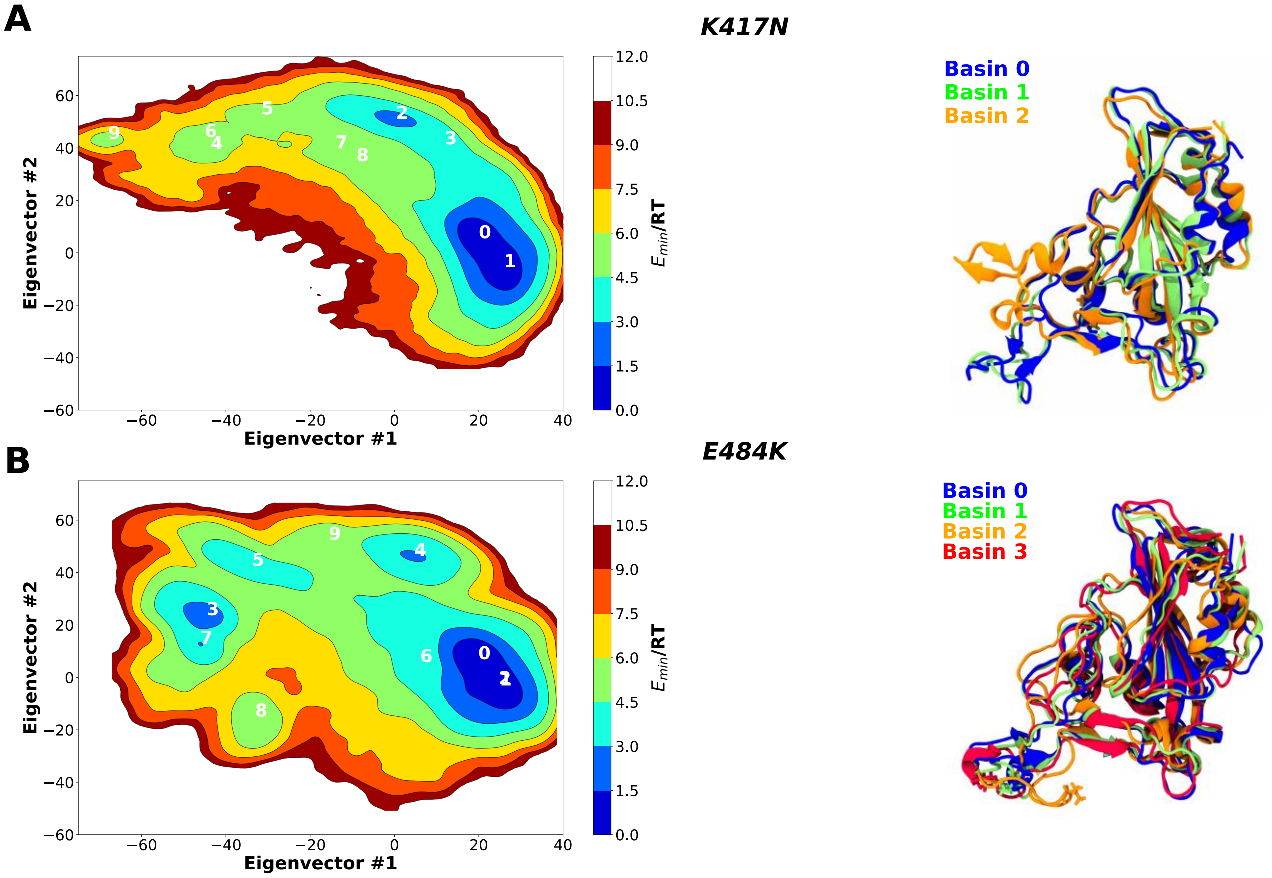

Figure S3. Two-dimension principal component analysis (PCA) of SARS-CoV-2 RBD's mutants conformational dynamics in water. Plots of the first two principal components determined from the Cα backbone of the (A) K417N and (B) E484K RBD mutants. Snapshots of the lowest energy structures for selected basins are also shown.

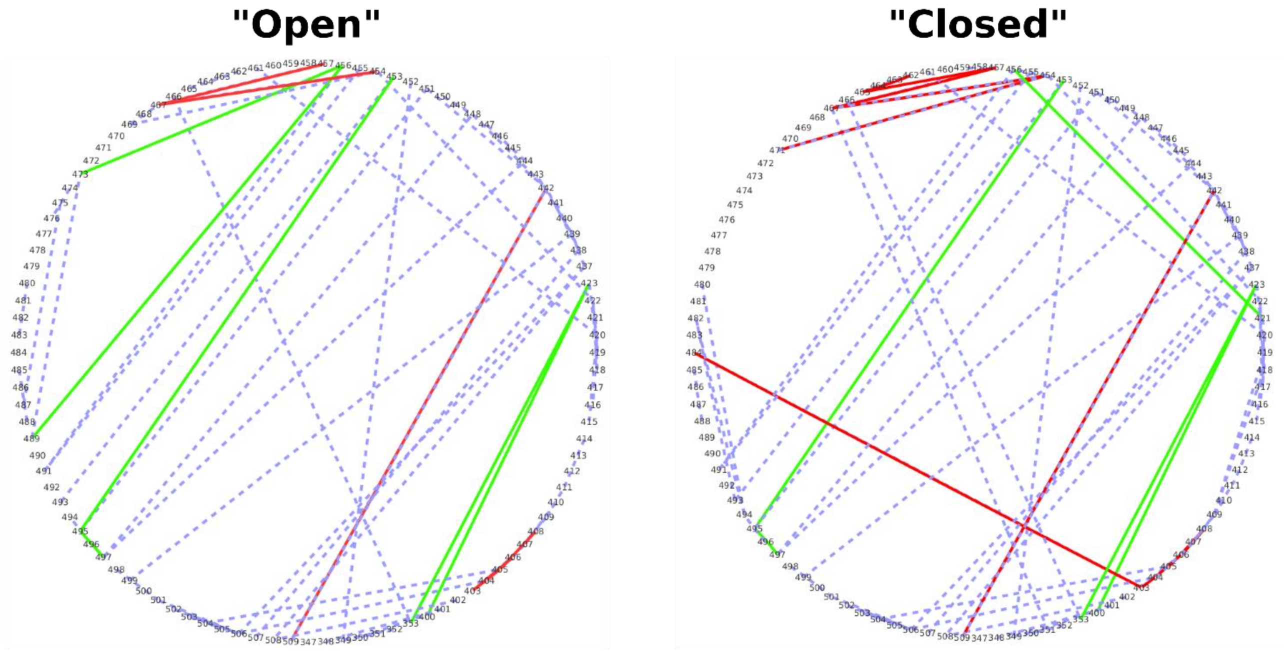

Figure S4. Residue interaction networks (RINs) for the "open" and "closed" conformations of *wt* RBD. RINs determined using RIP-MD for the 5000 lowest energy conformations obtained for the most populated "open" and "closed" basins of *wt* RBD. Hydrogen bonds, salt bridges and pi-pi interactions are shown in blue, red and green, respectively.

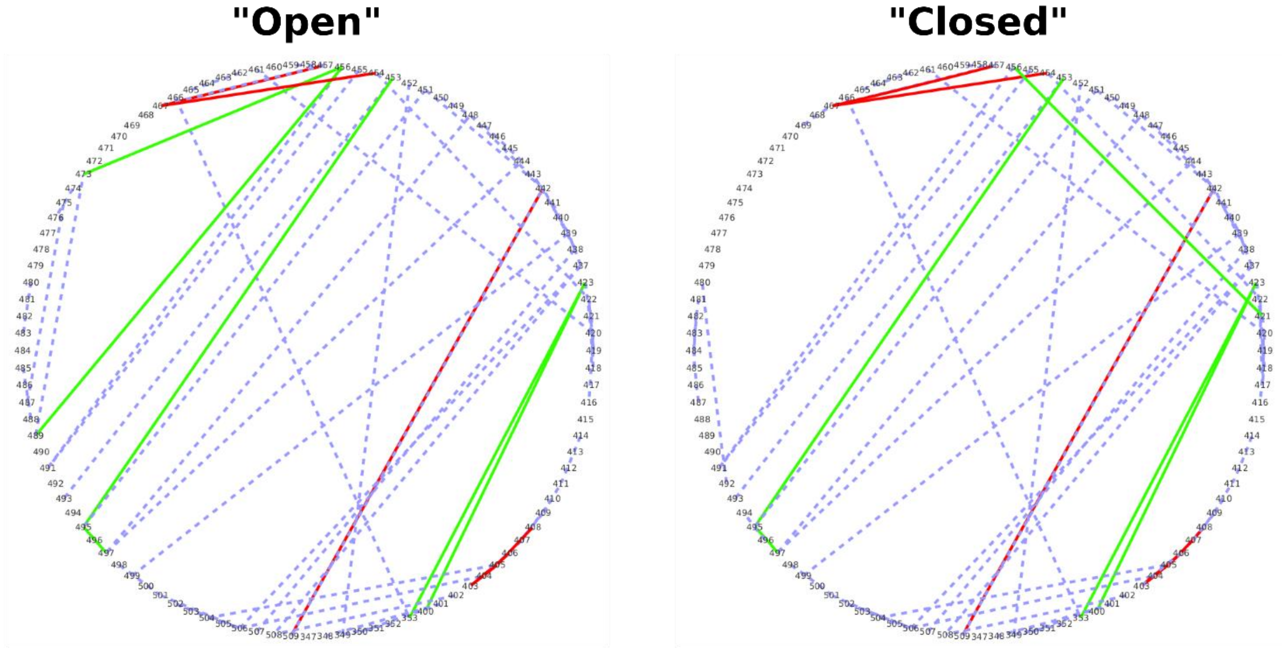

Figure S5. Residue interaction networks (RINs) for the "open" and "closed" conformations of the alpha variant RBD. RINs determined using RIP-MD for the 5000 lowest energy conformations obtained for the most populated "open" and "closed" basins of alpha RBD. Hydrogen bonds, salt bridges and pi-pi interactions are shown in blue, red and green, respectively.

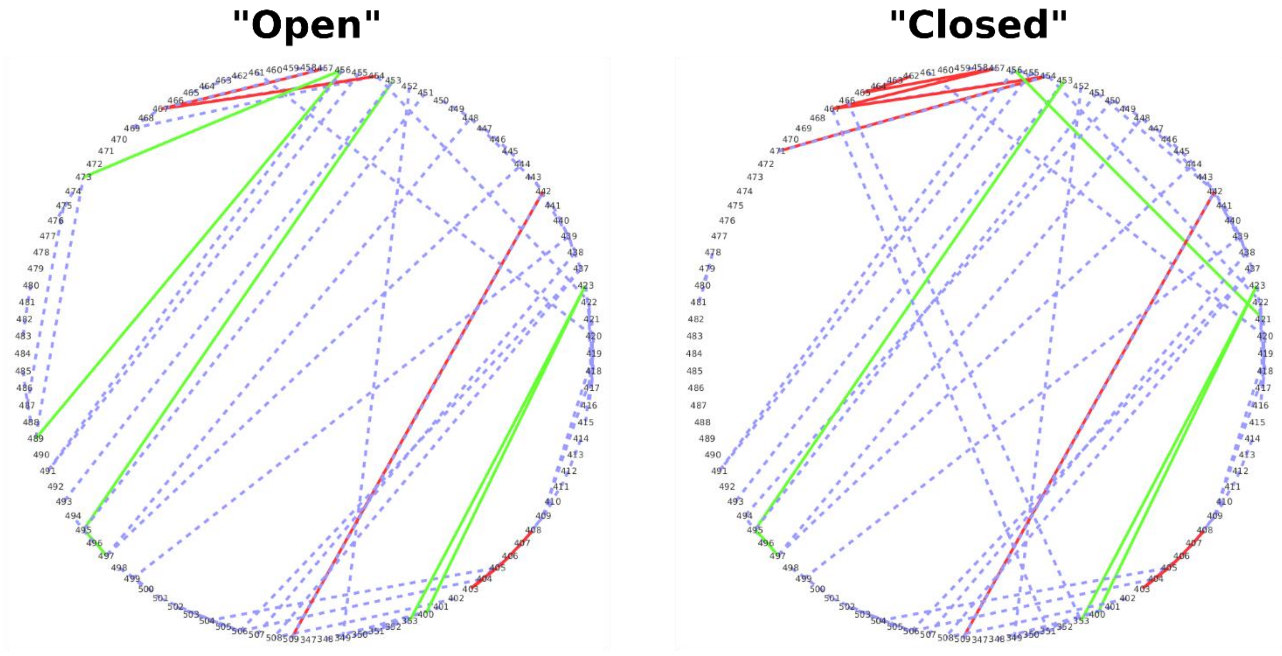

Figure S6. Residue interaction networks (RINs) for the “open” and “closed” conformations of the beta variant RBD. RINs determined using RIP-MD for the 5000 lowest energy conformations obtained for the most populated “open” and “closed” basins of beta RBD. Hydrogen bonds, salt bridges and pi-pi interactions are shown in blue, red and green, respectively.

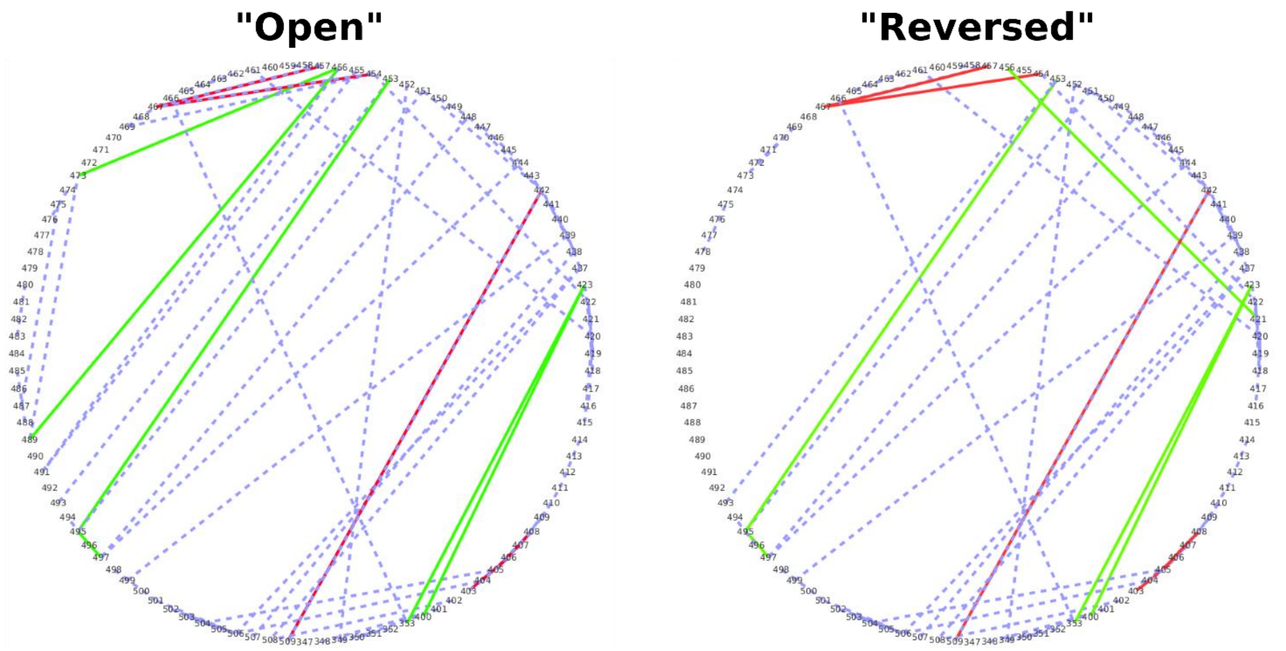

Figure S7. Residue interaction networks (RINs) for the “open” and “reversed” conformations of the delta variant RBD. RINs determined using RIP-MD for the 5000 lowest energy conformations obtained for “open” and “reversed” basins of delta RBD. Hydrogen bonds, salt bridges and pi-pi interactions are shown in blue, red and green, respectively.

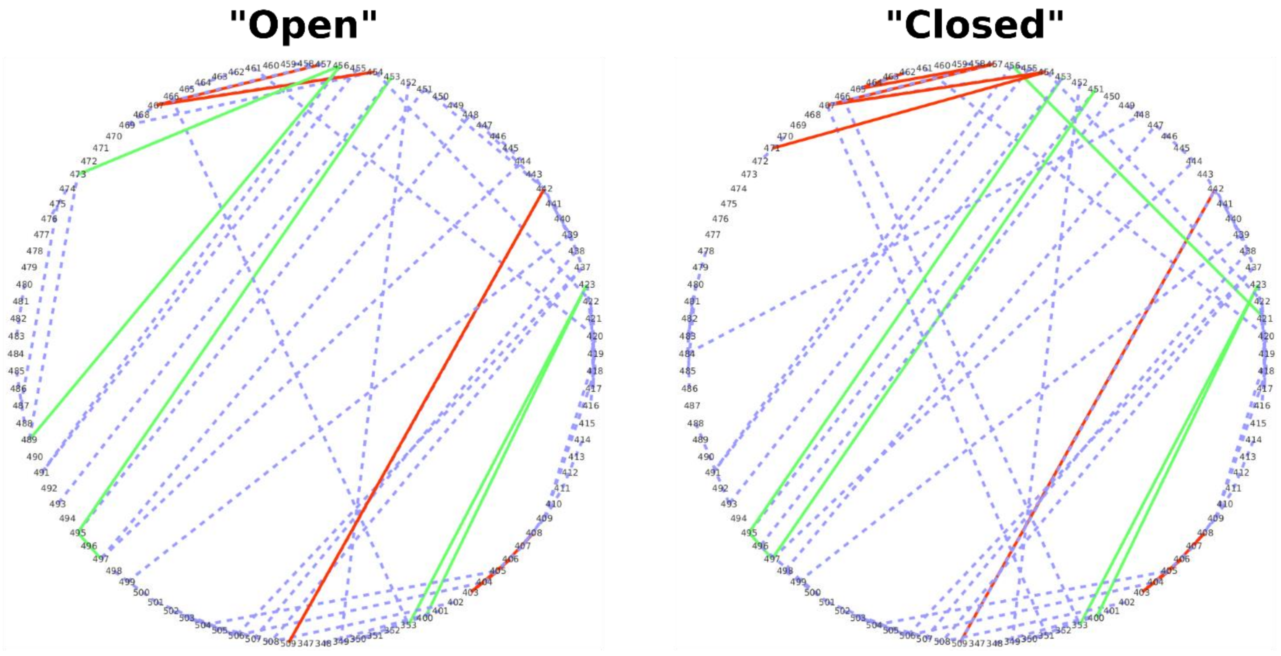

Figure S8. Residue interaction networks (RINs) for the “open” and “reversed” conformations of the omicron variant RBD. RINs determined using RIP-MD for the 5000 lowest energy conformations obtained for “open” and “reversed” basins of omicron RBD. Hydrogen bonds, salt bridges and pi-pi interactions are shown in blue, red and green, respectively.

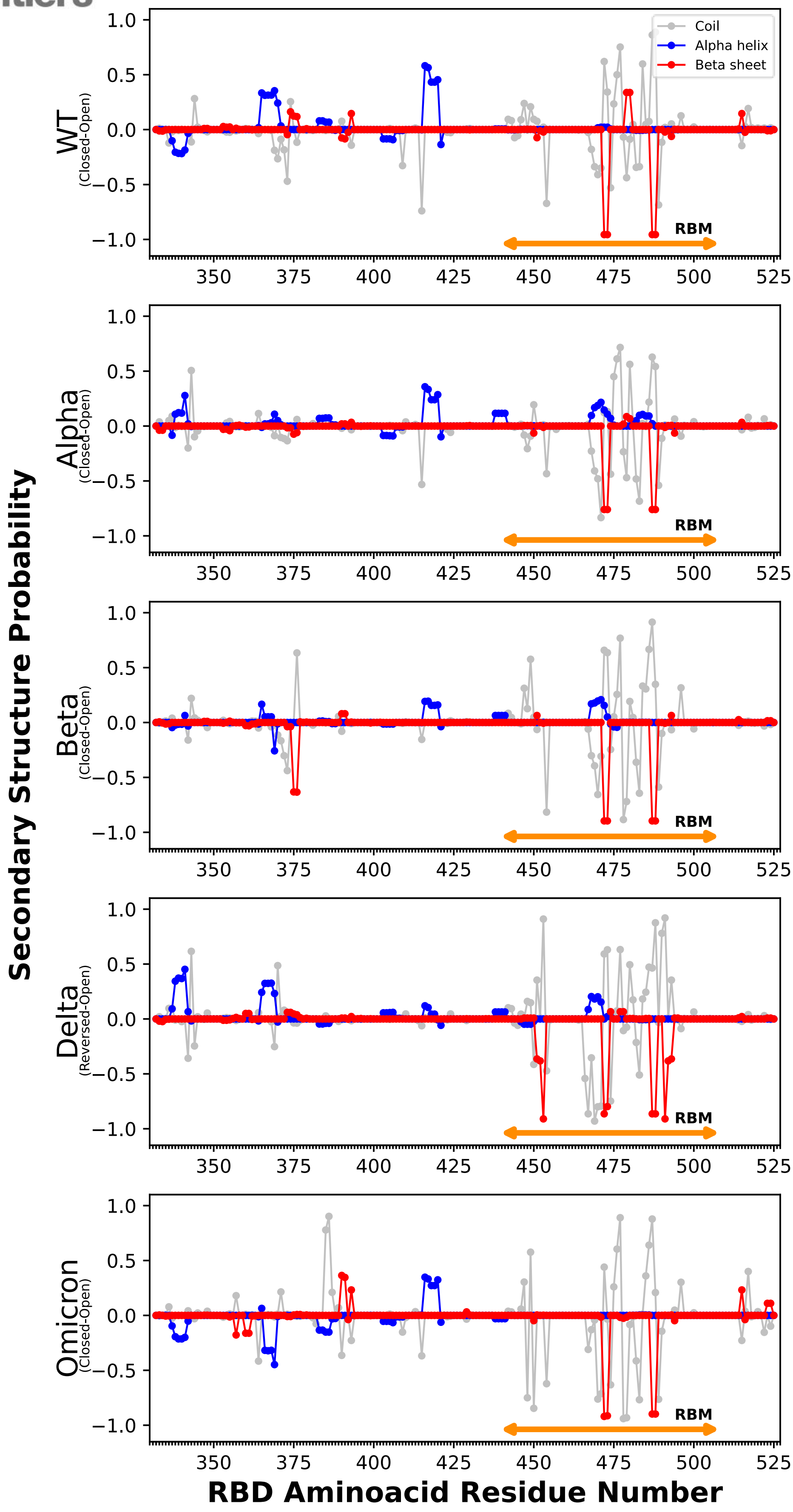

Figure S9. Secondary structure difference between closed and open conformations of *wt*, alpha, beta, omicron and delta SARS-CoV-2 RBD simulated in water. Probability of coil, α-helix and β-sheet secondary structures was obtained using the GROMACS tool gmx do_dssp(1) for all conformations (open, closed and reversed) of all four variants. RBM residues are shown with an orange arrow.

Table S2. Surface Accessible Surface Area (SASA) analysis of SARS-CoV-2 RBD in water. SASA values were calculated using the GROMACS tool gmx_sasa(1) for the whole trajectory (Entire trajectory) and for the two major conformations ("open"/"closed " and "open"/"reversed"). Results were also divided in the contribution of hydrophobic atoms, which are the ones with charges [-0.2, 0.2], and hydrophilic, those outside of this range. 95 % Confidence intervals (CI) were calculated with bootstrap resampling.

Variant	Protein SASA (nm^2)	Hydrophobic atoms SASA (nm^2)	Hydrophilic atoms SASA (nm^2)
	Average ± CI (95%)	Average ± CI (95%)	Average ± CI (95%)
WT			
Average	$107.72^{+0.07}_{-0.07}$	$55.07^{+0.05}_{-0.05}$	$52.62^{+0.05}_{-0.05}$
"Open"	$109.08^{+0.05}_{-0.05}$	$55.11^{+0.04}_{-0.04}$	$53.97^{+0.04}_{-0.05}$
"Closed"	$105.52^{+0.05}_{-0.05}$	$53.68^{+0.04}_{-0.04}$	$51.57^{+0.04}_{-0.04}$
**Alpha**			
Average	$108.16^{+0.04}_{-0.04}$	$55.13^{+0.03}_{-0.03}$	$53.04^{+0.03}_{-0.03}$
"Open"	$108.62^{+0.06}_{-0.06}$	$55.26^{+0.04}_{-0.04}$	$53.36^{+0.04}_{-0.04}$
"Closed"	$102.99^{+0.07}_{-0.07}$	$52.69^{+0.04}_{-0.04}$	$50.31^{+0.05}_{-0.05}$
**Beta**			
Average	$108.10^{+0.01}_{-0.01}$	$54.95^{+0.01}_{-0.01}$	$53.15^{+0.01}_{-0.01}$
"Open"	$108.58^{+0.06}_{-0.06}$	$53.70^{+0.04}_{-0.04}$	$53.88^{+0.04}_{-0.04}$
"Closed"	$107.65^{+0.08}_{-0.08}$	$56.32^{+0.05}_{-0.05}$	$51.33^{+0.05}_{-0.06}$
**Delta**			
Average	$109.62^{+0.11}_{-0.11}$	$55.46^{+0.08}_{-0.08}$	$54.16^{+0.08}_{-0.07}$
"Open"	$109.58^{+0.05}_{-0.05}$	$54.87^{+0.04}_{-0.04}$	$54.70^{+0.04}_{-0.04}$
"Reversed"	$106.08^{+0.07}_{-0.07}$	$53.65^{+0.04}_{-0.04}$	$52.43^{+0.04}_{-0.04}$
**Omicron**			
Average	$110.97^{+0.04}_{-0.04}$	$56.84^{+0.03}_{-0.03}$	$54.13^{+0.02}_{-0.02}$
"Open"	$110.83^{+0.05}_{-0.05}$	$56.68^{+0.04}_{-0.04}$	$54.16^{+0.04}_{-0.04}$
"Closed"	$109.73^{+0.07}_{-0.07}$	$57.07^{+0.07}_{-0.07}$	$52.66^{+0.05}_{-0.05}$

Table S3. Compilation of ACE2-RBD binding kinetics data from recent studies. Kinetic parameters of ACE2 binding to *wt*, alpha, beta, delta and omicron RBD/Spike variants data obtained from SPR and BLI(2,3,12,13,4–11).

			WT			Alpha			Beta			Delta			Omicron		
Reference	RBD/Spike	Technique	K_{on} ($M^{-1} s^{-1}$)	K_{off} (s^{-1})	K_d (nM)	K_{on} ($M^{-1} s^{-1}$)	K_{off} (s^{-1})	K_d (nM)	K_{on} ($M^{-1} s^{-1}$)	K_{off} (s^{-1})	K_d (nM)	K_{on} ($M^{-1} s^{-1}$)	K_{off} (s^{-1})	K_d (nM)	K_{on} ($M^{-1} s^{-1}$)	K_{off} (s^{-1})	K_d (nM)
McCallum et al. 2021 [2]	RBD	SPR	7.70×10^{4}	6.70×10^{-3}	78	7.50×10^{4}	1.20×10^{-3}	15	-	-	-	5.90×10^{4}	4.30×10^{-3}	63	-	-	-
Tian et al. 2021 [3]	RBD	SPR	2.50×10^{4}	2.10×10^{-4}	8.3	7.50×10^{4}	0.37×10^{-4}	0.5	6.40×10^{4}	0.30×10^{-4}	0.5	-	-	-	-	-	-
Laffeber et al. 2021 [4]	RBD	SPR	4.50×10^{5}	7.80×10^{-3}	17	5.70×10^{5}	1.30×10^{-3}	2.4	7.60×10^{5}	4.30×10^{-3}	5.8	-	-	-	-	-	-
Supasa et al. 2021 [5]	RBD	SPR	3.88×10^{4}	2.92×10^{-3}	75.1	6.38×10^{4}	6.85×10^{-4}	10.7	-	-	-	-	-	-	-	-	-
Wirnsberger et al. 2021 [6]	RBD	SPR	6.86×10^{5}	11.0×10^{-3}	16.2	4.36×10^{5}	1.59×10^{-3}	3.72	6.86×10^{5}	4.42×10^{-3}	6.5	10.1×10^{5}	8.23×10^{-3}	8.61	-	-	-
de Souza et al. 2021 [7]	RBD	SPR	0.90×10^{6}	91.6×10^{-4}	10.3	1.30×10^{6}	15.5×10^{-4}	1.2	1.20×10^{6}	39.4×10^{-4}	3.3	-	-	-	-	-	-
Lan et al. 2022 [8]	RBD	SPR	1.24×10^{6}	7.35×10^{-3}	59	-	-	-	-	-	-	-	-	-	1.73×10^{6}	4.86×10^{-3}	28
Dejnirattisai et al. 2022 [9]	RBD	SPR	7.00×10^{5}	5.10×10^{-3}	7.3	1.10×10^{6}	1.60×10^{-3}	1.5	1.30×10^{6}	4.00×10^{-3}	3.2	1.20×10^{6}	5.80×10^{-3}	4.9	8.20×10^{5}	6.40×10^{-3}	7.8
Meng et al. 2022 [10]	RBD	BLI	1.10×10^{5}	1.40×10^{-2}	127	-	-	-	-	-	-	5.90×10^{4}	1.10×10^{-2}	190	1.10×10^{5}	4.70×10^{-3}	44
Junker et al. 2022 [11]	RBD	BLI	1.26×10^{5}	1.72×10^{-4}	1.26	-	-	-	-	-	-	1.24×10^{5}	1.19×10^{-4}	0.96	1.85×10^{5}	1.45×10^{-4}	0.78
de Souza et al. 2021 [7]	Spike	SPR	0.09×10^{6}	5.80×10^{-4}	6.4	0.10×10^{6}	1.70×10^{-4}	0.1	0.30×10^{6}	3.00×10^{-4}	0.3	-	-	-	-	-	-
Saville et al. 2021 [12]	Spike	BLI	1.40×10^{5}	7.09×10^{-4}	5.06	-	-	-	-	-	-	1.51×10^{5}	4.01×10^{-4}	2.65	-	-	-
Yang et al. 2021 [13]	Spike	BLI	3.43×10^{4}	10.6×10^{-5}	3.1	3.86×10^{4}	5.25×10^{-5}	1.36	7.32×10^{4}	1.79×10^{-5}	0.25	4.39×10^{4}	1.71×10^{-5}	0.39	-	-	-

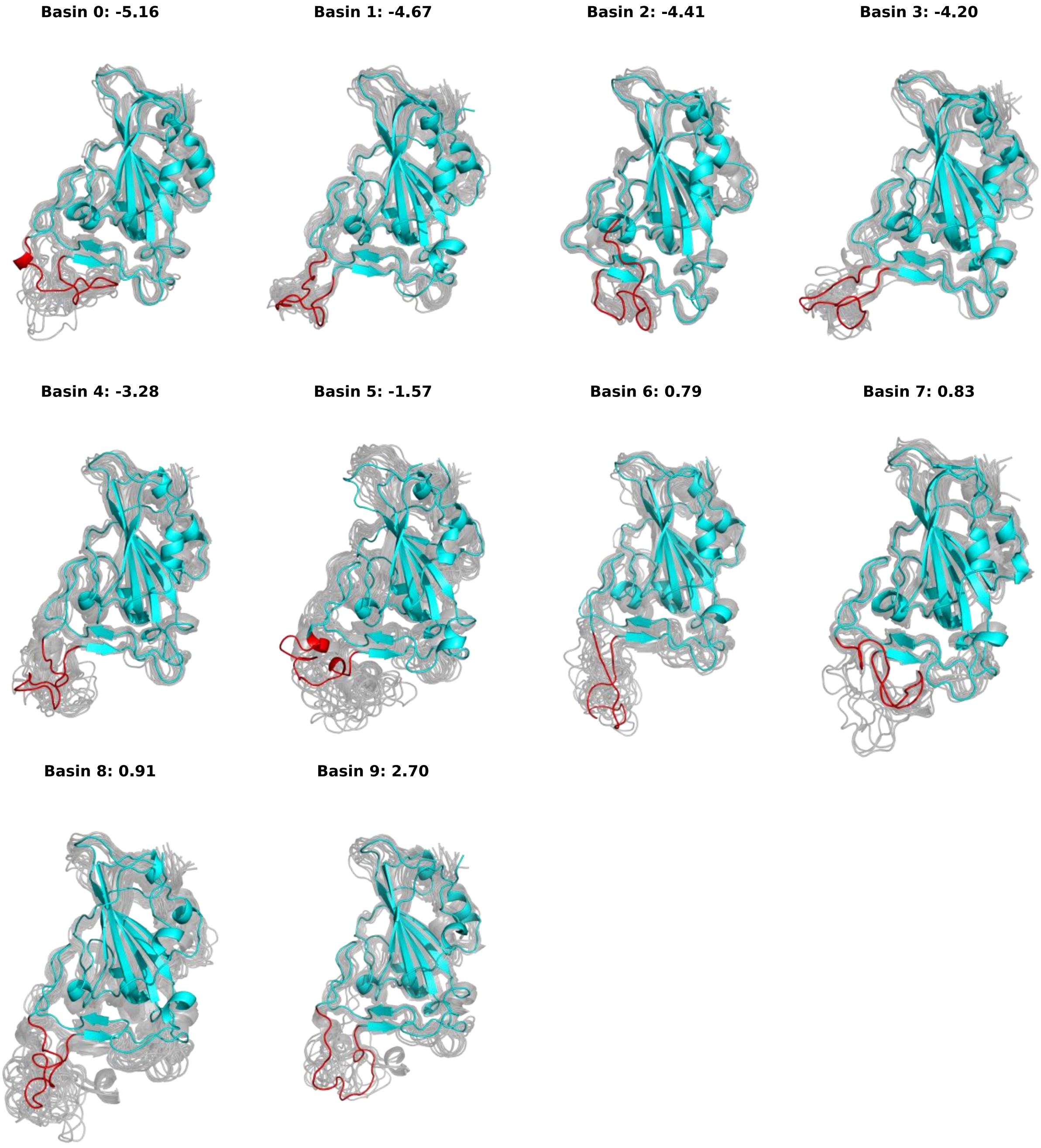

Figure S10. Snapshots representative of all *wt* RBD PCA basins. The structures corresponding to the free energy minima of all conformational basins are represented in blue, with the ridge region highlighted in red, together with structures sampled from the same basin (background, gray colored).

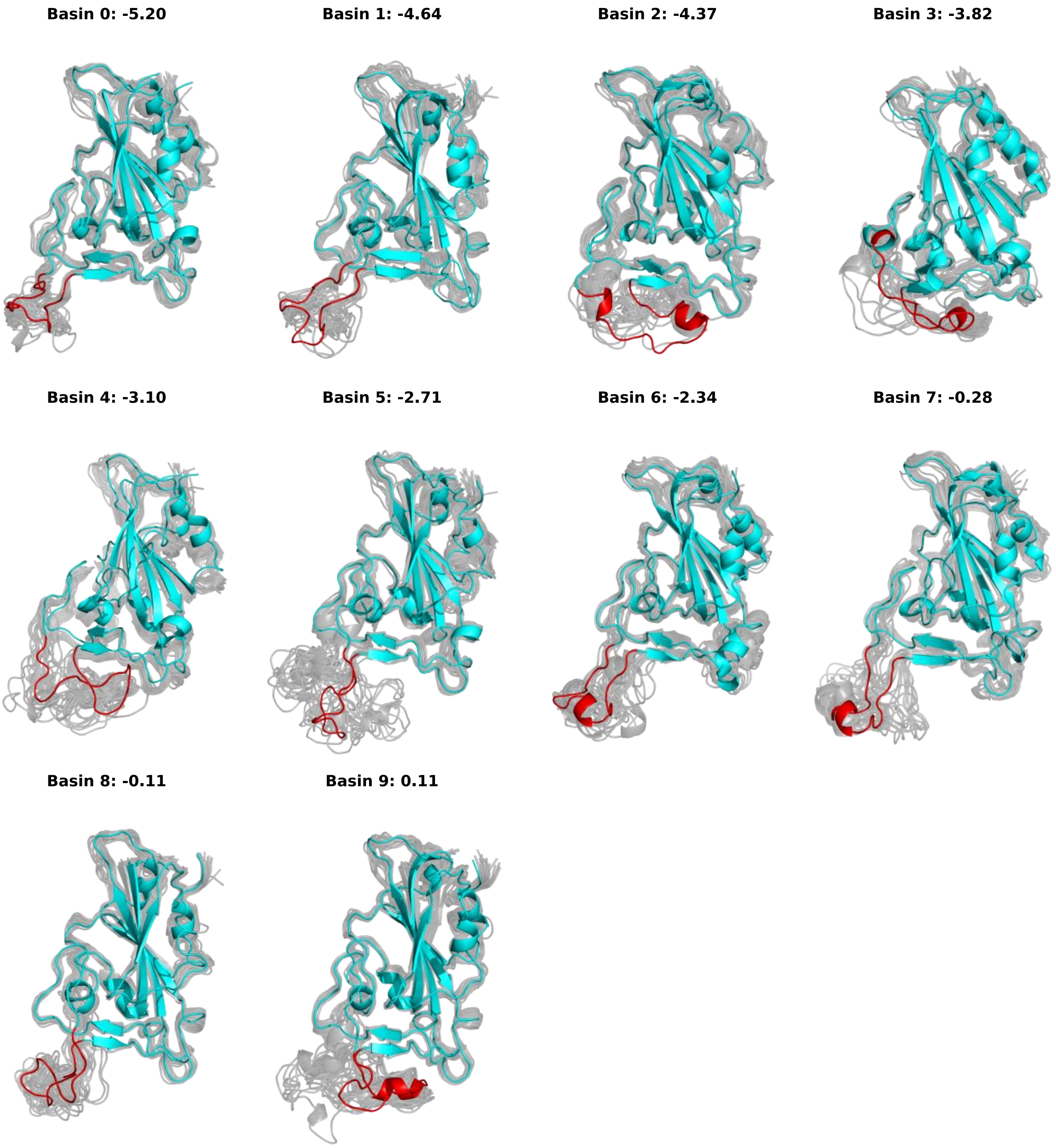

Figure S11. Structures representative of all alpha RBD PCA basins. The structures corresponding to the free energy minima of all conformational basins are represented in blue, with the ridge region highlighted in red, together with structures sampled from the same basin (background, gray colored).

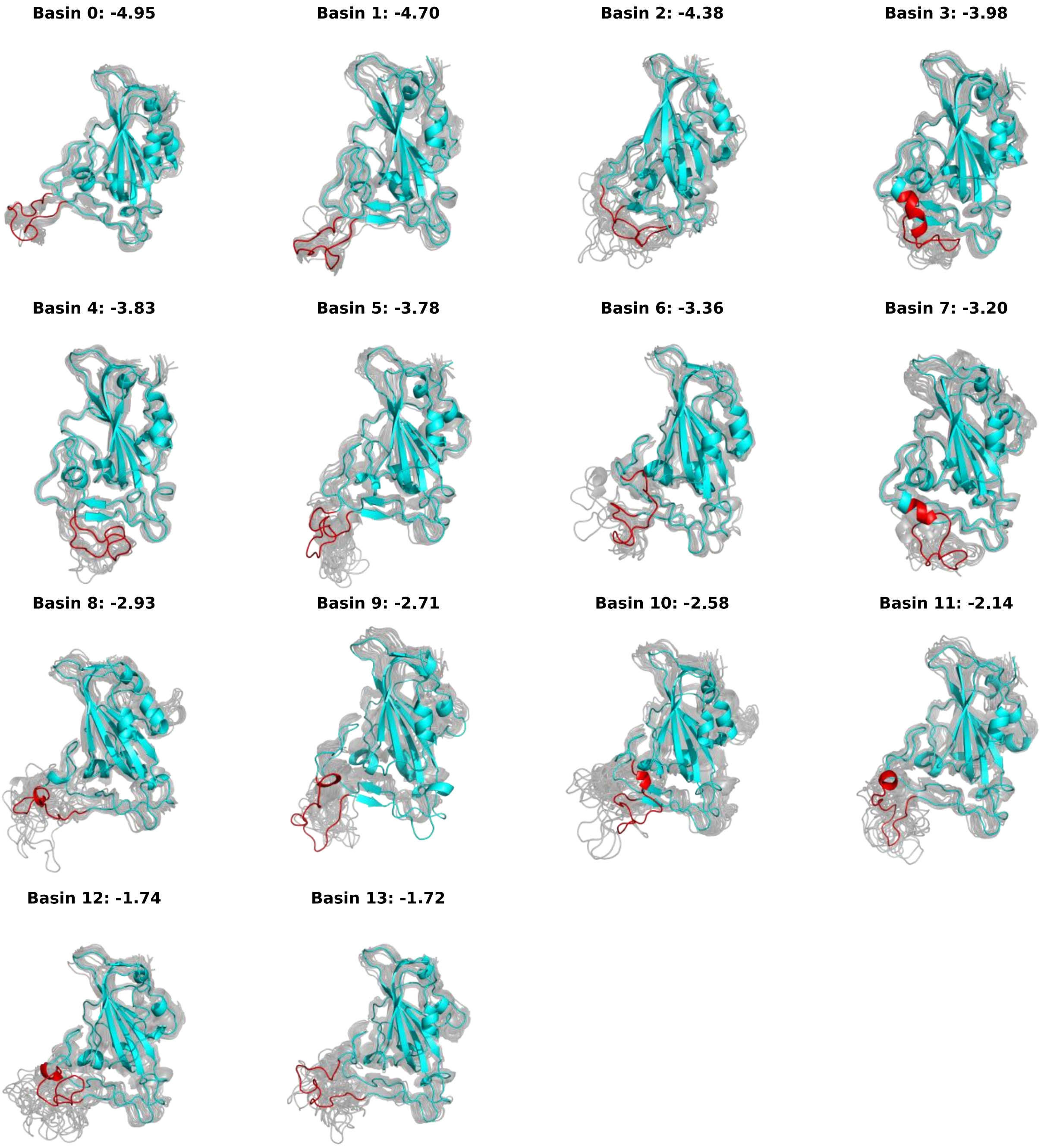

Figure S12. Structures representative of all beta RBD PCA basins. The structures corresponding to the free energy minima of all conformational basins are represented in blue, with the ridge region highlighted in red, together with structures sampled from the same basin (background, gray colored).

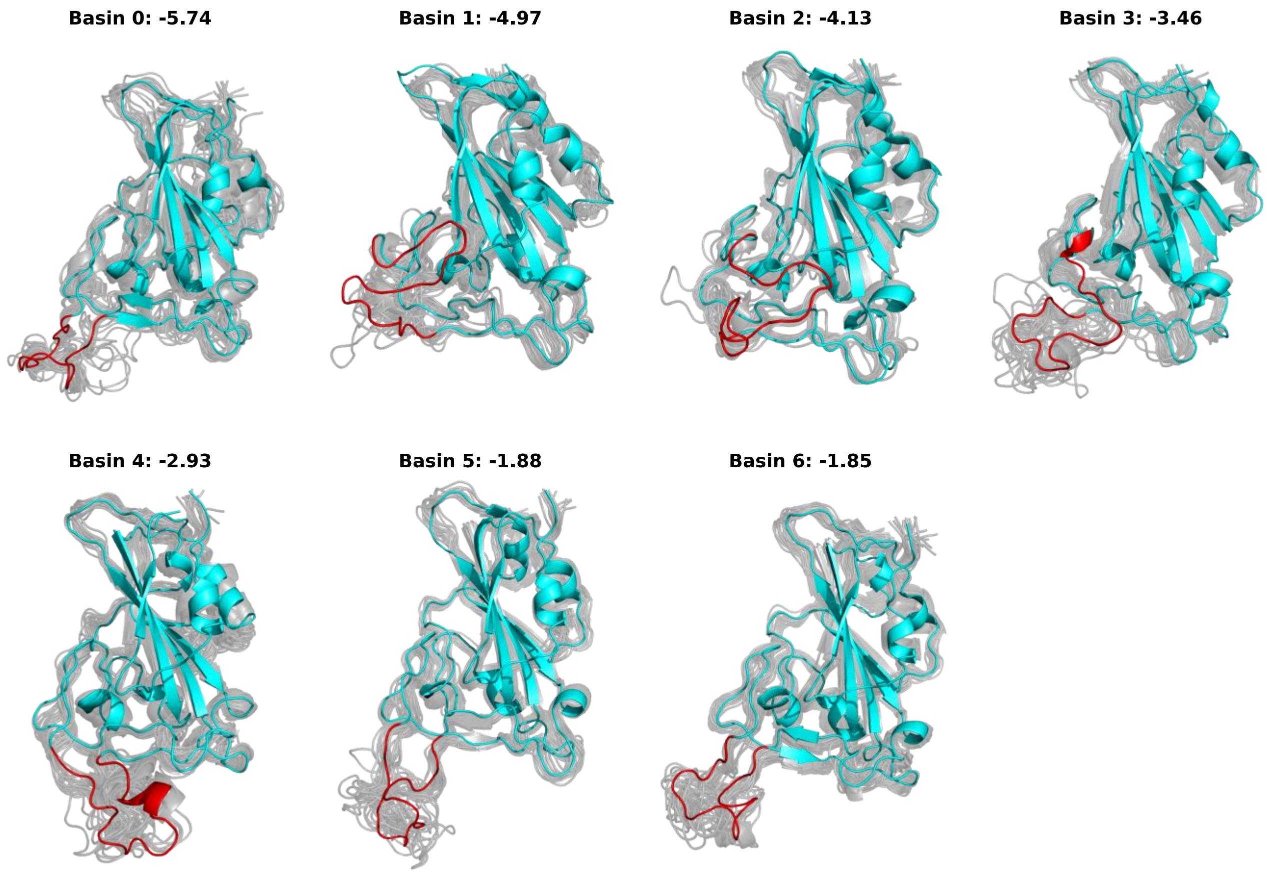

Figure S13. Structures representative of all delta RBD PCA basins. The structures corresponding to the free energy minima of all conformational basins are represented in blue, with the ridge region highlighted in red, together with structures sampled from the same basin (background, gray colored).

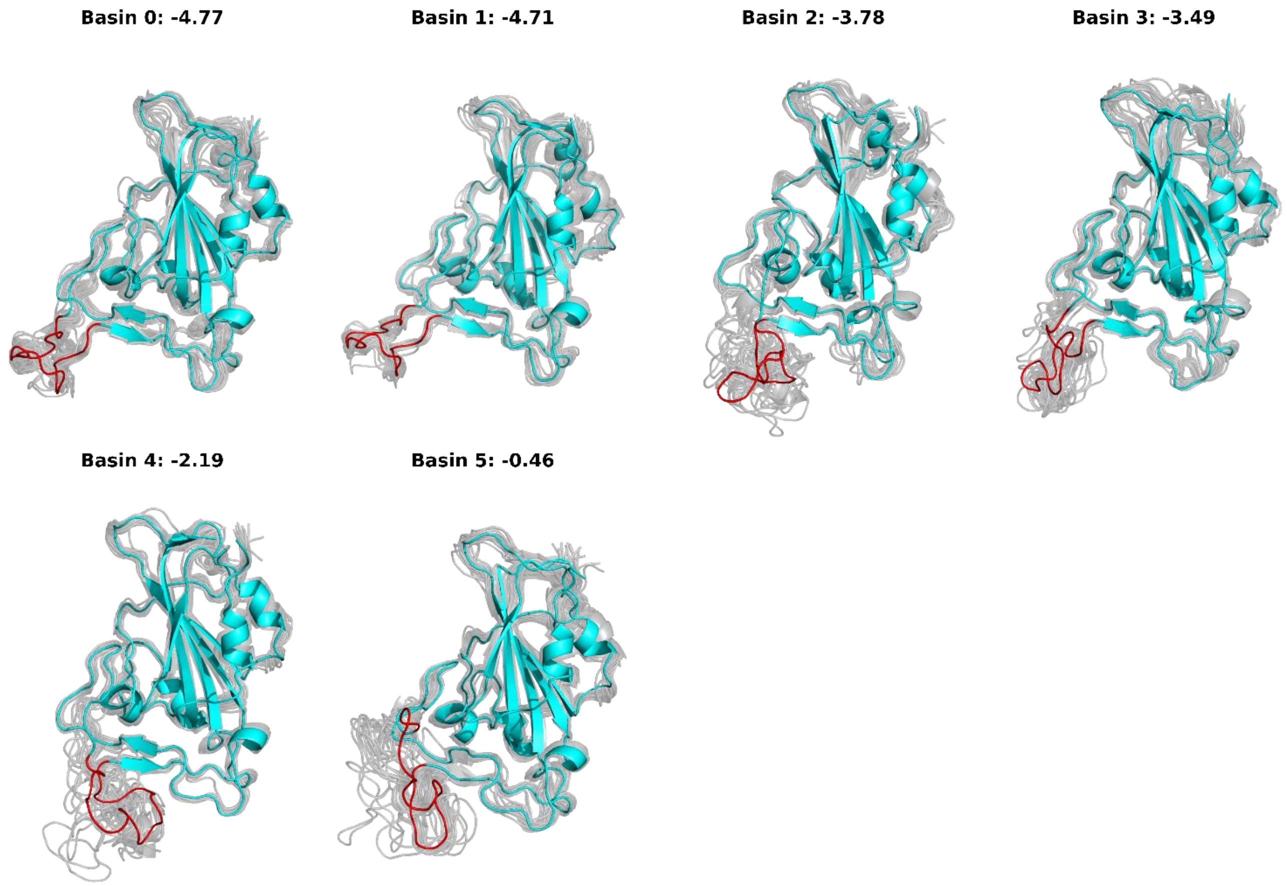

Figure S14. Structures representative of all omicron RBD PCA basins. The structures corresponding to the free energy minima of all conformational basins are represented in blue, with the ridge region highlighted in red, together with structures sampled from the same basin (background, gray colored).

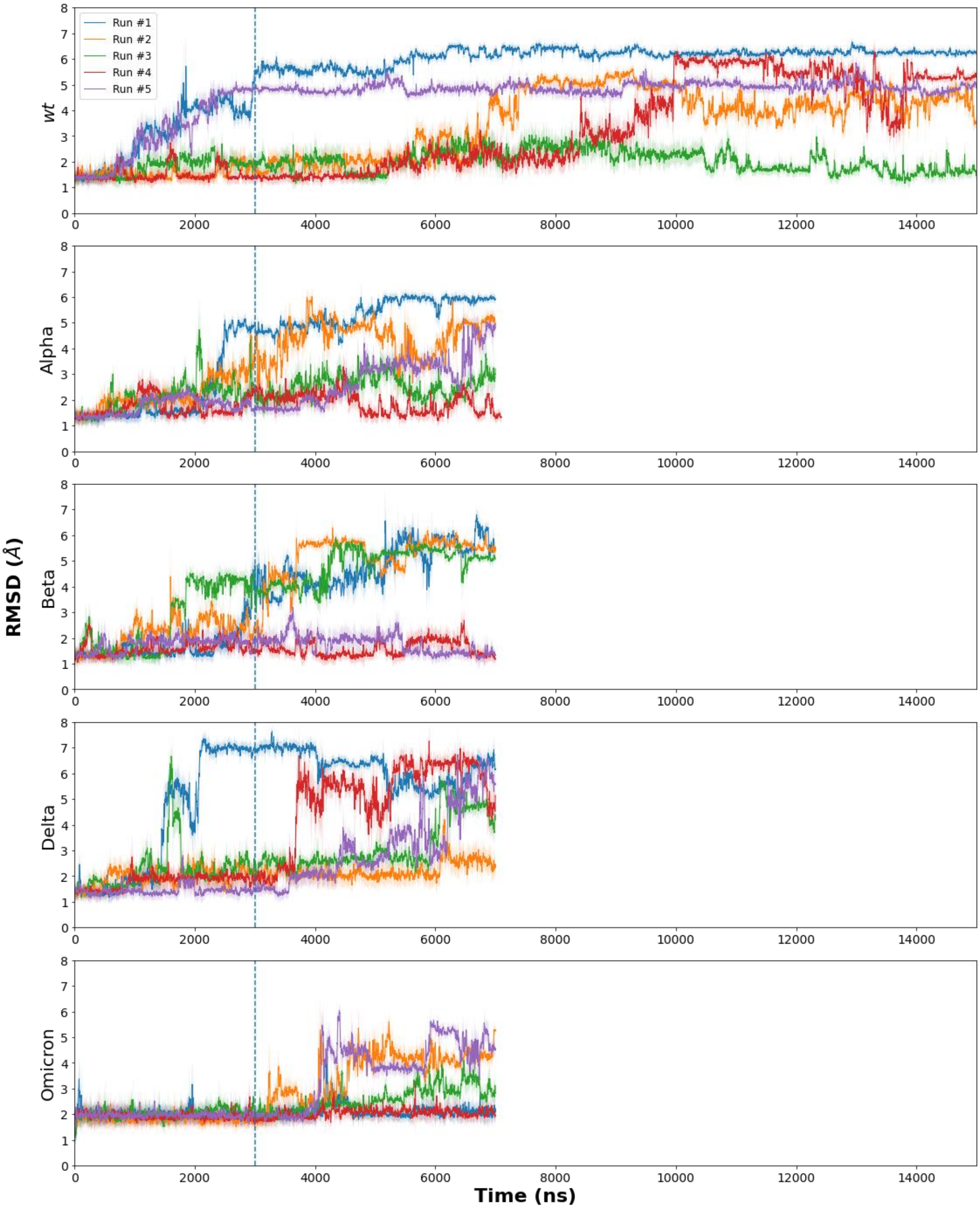

Figure S15. RBD Cα root-mean-square deviation (RMSD) moving average in solution. Data shown for the five replicas for each variant tested. Cα were fitted against the RBD X-ray structure from PDB ID: 6M0J. The moving average was calculated using the neighboring 50 frames. The first 3 μs of simulation were used for equilibration (blue dashed line) and the remaining frames were used for further PCA and RIN analysis.

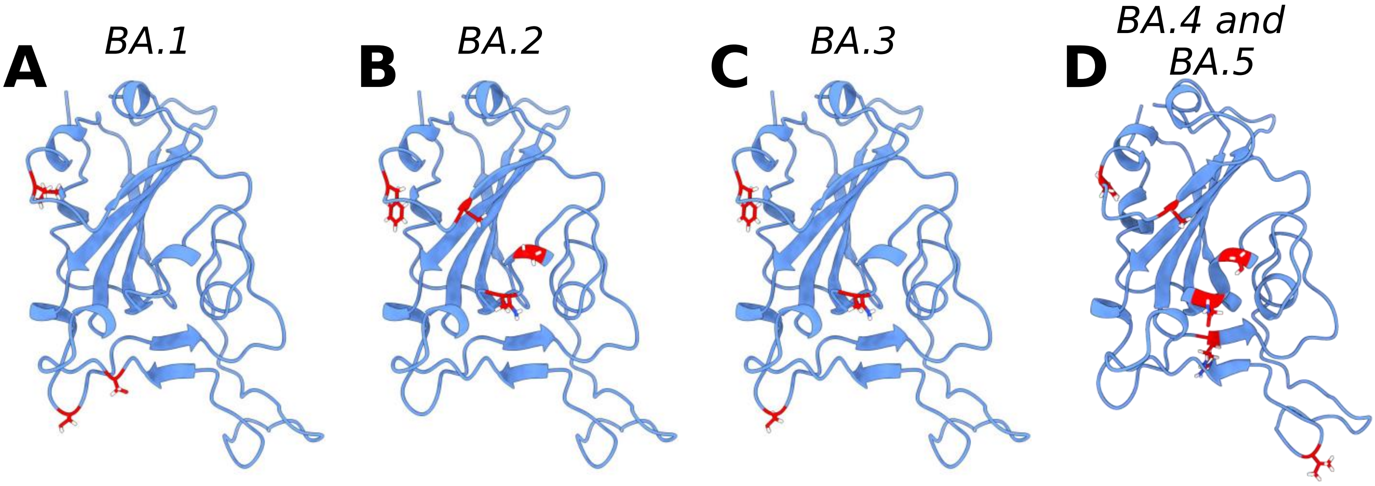

Figure S16. Representation of the RBD of the three Omicron lineages and their specific mutations. The RBD regions of all three lineages are shown side-by-side in blue. Specific mutations of lineages (A) BA.1 [S371L, G446S, G496S], (B) BA.2 [S371F, T376A, D405N, R408S], (C) BA.3 [S371F, D405N, G446S] and (D) BA.4 and BA.5 [S371F, T376A, D405N, R408S, L452R, F486V] are shown in sticks and highlighted in red.

References

1. Abraham MJ, Murtola T, Schulz R, Páll S, Smith JC, Hess B, et al. Gromacs: High performance molecular simulations through multi-level parallelism from laptops to supercomputers. SoftwareX. 2015 Sep 1;1–2:19–25.

2. McCallum M, Walls AC, Sprouse KR, Bowen JE, Rosen LE, Dang H V, et al. Molecular basis of immune evasion by the Delta and Kappa SARS-CoV-2 variants. Science [Internet]. 2021 Aug 12 [cited 2021 Oct 3];eabl8506. Available from: http://www.ncbi.nlm.nih.gov/pubmed/34751595

3. Tian F, Tong B, Sun L, Shi S, Zheng B, Wang Z, et al. N501y mutation of spike protein in sars-cov-2 strengthens its binding to receptor ace2. Elife. 2021 Aug 20;10.

4. Laffeber C, de Koning K, Kanaar R, Lebbink JHG. Experimental Evidence for Enhanced Receptor Binding by Rapidly Spreading SARS-CoV-2 Variants. J Mol Biol. 2021 Jul 23;433(15):167058.

5. Supasa P, Zhou D, Dejnirattisai W, Liu C, Mentzer AJ, Ginn HM, et al. Reduced neutralization of SARS-CoV-2 B.1.1.7 variant by convalescent and vaccine sera. Cell [Internet]. 2021 Apr 15 [cited 2021 Oct 3];184(8):2201-2211.e7. Available from: http://www.cell.com/article/S0092867421002221/fulltext

6. Wirnsberger G, Monteil V, Eaton B, Postnikova E, Murphy M, Braunsfeld B, et al. Clinical grade ACE2 as a universal agent to block SARS-CoV-2 variants. bioRxiv [Internet]. 2021 Sep 10 [cited 2021 Oct 3];2021.09.10.459744. Available from: https://www.biorxiv.org/content/10.1101/2021.09.10.459744v1%0Ahttps://www.biorxiv.org/content/10.1101/2021.09.10.459744v1.abstract

7. Souza AS de, Amorim VM de F, Guardia GDA, Santos FRC dos, Santos FF dos, Souza RF de, et al. Molecular dynamics analysis of fast-spreading severe acute respiratory syndrome coronavirus 2 variants and their effects in the interaction with human angiotensin-converting enzyme 2. bioRxiv [Internet]. 2021 Jun 15 [cited 2021 Oct 3];2021.06.14.448436. Available from: https://www.biorxiv.org/content/10.1101/2021.06.14.448436v1%0Ahttps://www.biorxiv.org/content/10.1101/2021.06.14.448436v1.abstract

8. Lan J, He X, Ren Y, Wang Z, Zhou H, Fan S, et al. Structural and computational insights into the SARS-CoV-2 Omicron RBD-ACE2 interaction. bioRxiv [Internet]. 2022 Jan 4 [cited 2022 Mar 20];2022.01.03.474855. Available from: https://www.biorxiv.org/content/10.1101/2022.01.03.474855v1

9. Dejnirattisai W, Huo J, Zhou D, Zahradník J, Supasa P, Liu C, et al. SARS-CoV-2 Omicron-B.1.1.529 leads to widespread escape from neutralizing antibody responses. Cell. 2022 Feb 3;185(3):467-484.e15.

10. Meng B, Abdullahi A, Ferreira IATM, Goonawardane N, Saito A, Kimura I, et al. Altered TMPRSS2 usage by SARS-CoV-2 Omicron impacts infectivity and fusogenicity. Nat 2022 [Internet]. 2022 Feb 1 [cited 2022 Mar 20];1–9. Available from: https://www.nature.com/articles/s41586-022-04474-x

11. Junker D, Becker M, Wagner TR, Kaiser PD, Maier S, Grimm TM, et al. Antibody binding and ACE2 binding inhibition is significantly reduced for the Omicron variant compared to all other variants of concern. medRxiv [Internet]. 2022 Jan 1 [cited 2022

Mar 20];6(8):2021.12.30.21267519. Available from: https://www.medrxiv.org/content/10.1101/2021.12.30.21267519v1

12. Saville JW, Mannar D, Zhu X, Srivastava SS, Berezuk AM, Demers J-P, et al. Structural and Biochemical Rationale for Enhanced Spike Protein Fitness in Delta and Kappa SARS-CoV-2 Variants. bioRxiv [Internet]. 2021 Sep 2 [cited 2021 Oct 3];2021.09.02.458774. Available from: https://www.biorxiv.org/content/10.1101/2021.09.02.458774v1%0Ahttps://www.biorxiv.org/content/10.1101/2021.09.02.458774v1.abstract

13. Yang T-J, Yu P-Y, Chang Y-C, Chang N-E, Tsai Y-X, Liang K-H, et al. Structure-activity relationships of B.1.617 and other SARS-CoV-2 spike variants. bioRxiv [Internet]. 2021 Sep 13 [cited 2022 Mar 20];2021.09.12.459978. Available from: https://www.biorxiv.org/content/10.1101/2021.09.12.459978v1
